# Galaninergic and hypercapnia-activated neuronal projections to the ventral respiratory column

**DOI:** 10.1007/s00429-024-02782-8

**Published:** 2024-04-05

**Authors:** Ayse S. Dereli, Alice Y. S. Oh, Simon McMullan, Natasha N. Kumar

**Affiliations:** 1 Department of Pharmacology, School of Biomedical Sciences, University of New South Wales, Sydney, Australia; 2 Macquarie Medical School, Macquarie University, Sydney, Australia

**Keywords:** Galanin, Ventral respiratory column, Neuronal tracing, Breathing regulation, Central chemoreception, c-Fos

## Abstract

In mammals, the ventral respiratory column (VRC) plays a pivotal role in integrating neurochemically diverse inputs from brainstem and forebrain regions to generate respiratory motor patterns. VRC microinjection of the neuropeptide galanin has been reported to dampen carbon dioxide (CO_2_)-mediated chemoreflex responses. Additionally, we previously demonstrated that galaninergic neurons in the retrotrapezoid nucleus (RTN) are implicated in the adaptive response to hypercapnic stimuli, suggesting a link between RTN neuroplasticity and increased neuronal drive to the VRC. VRC neurons express galanin receptor 1, suggesting potential regulatory action by galanin, however, the precise galaninergic chemoreceptor-VRC circuitry remains to be determined. This study aimed to identify sources of galaninergic input to the VRC that contribute to central respiratory chemoreception. We employed a combination of retrograde neuronal tracing, in situ hybridisation and immunohistochemistry to investigate VRC-projecting neurons that synthesise galanin mRNA. In an additional series of experiments, we used acute hypercapnia exposure (10% CO_2_, 1 h) and c-Fos immunohistochemistry to ascertain which galaninergic nuclei projecting to the VRC are activated. Our findings reveal that a total of 30 brain nuclei and 51 subnuclei project to the VRC, with 12 of these containing galaninergic neurons, including the RTN. Among these galaninergic populations, only a subset of the RTN neurons (approximately 55%) exhibited activation in response to acute hypercapnia. Our findings highlight that the RTN is the likely source of galaninergic transmission to the VRC in response to hypercapnic stimuli.

**Supplementary Information:**

The online version contains supplementary material available at 10.1007/s00429-024-02782-8.

## Introduction

Located within the ventrolateral medulla, the ventral respiratory column (VRC) plays a crucial role in generating and regulating respiratory motor patterns. The VRC consists of four distinct compartments: Bötzinger Complex (BötC), preBötzinger Complex (preBötC), rostral ventral respiratory group (rVRG), and caudal ventral respiratory group (cVRG) (Guyenet et al. 2019). The BötC and preBötC together form the central respiratory pattern generator. Specifically, the preBötC is responsible for the intrinsic rhythmicity of inspiration (Feldman and Del Negro 2006; Koshiya and Smith 1999; Rekling and Feldman 1998; Smith et al. 2000, 1991), while the BötC is responsible for sculpting the expiratory phase of the respiratory cycle (Ezure 1990; Ezure et al. 2003; Jiang and Lipski 1990; Tian et al. 1999). This rhythmic activity is relayed to premotor neurons in the rVRG, which contains bulbospinal inspiratory excitatory neurons that are driven by the preBötC during inspiration and inhibited by BötC neurons during expiration (Smith et al. 2009), and the cVRG, which contains bulbospinal expiratory excitatory neurons, presumed to serve as the expiratory counterpart to the inspiratory rVRG (Smith et al. 2009).

Central respiratory pattern generation requires intricate neuronal interactions; both in vivo and in vitro voltage clamp techniques demonstrate that this mechanism relies on synaptic interaction, characterised by excitatory glutamatergic synaptic activation through AMPA and NMDA receptors. Whereas inhibitory GABA_A_, GABA_B_ and glycine receptor-mediated synaptic inputs sculpt the burst pattern (Busselberg et al. 2001; Richter et al. 1996).

Beyond the roles of fast transmitters in the respiratory central pattern generator, pharmacological and immunohistochemical studies have revealed that the VRC possesses receptors for a range of signalling molecules, including vasopressin (Kc et al. 2002), oxytocin (Mack et al. 2007), substance P (Gray et al. 1999; Langer et al. 2017b; Liu et al. 2004; Muere et al. 2015a, b), μ-opioid, (Gray et al. 1999; Langer et al. 2017a), 5-HT (Langer et al. 2017b; Muere et al. 2015b; Ptak et al. 2009), somatostatin (Le et al. 2016; Burke et al. 2010), neuromedin B (Li et al. 2016) gastrin releasing peptide (Li et al. 2016) and galanin (Dereli et al. 2019). Thus, various neurotransmitter systems are implicated in the regulation of breathing.

A combination of in vivo and in vitro local acidification, c-Fos immunochemistry, genetic investigations and pharmacological studies, showed that central chemoreception mechanisms modulate the respiratory behaviour. Specifically, the retrotrapezoid nucleus (RTN), nucleus tractus solitarius (NTS), raphe, periaqueductal grey area (PAG) and lateral parabrachial nucleus (LPB) contain carbon dioxide (CO_2_)-responsive neurons (Damasceno et al. 2014; Kaur et al. 2013; Nattie and Li 2012; Yokota et al. 2015). Focal acidification studies show that the RTN, NTS, locus coeruleus (LC) and dorsomedial hypothalamic nucleus (DMH) have increased firing rate in response to decrease in pH with an increase in ventilation (Nattie and Li 2002, 2010, 2012). Another body of work showed that these populations have increased c-Fos immunoreactivity in response to high levels of PaCO_2_ (Spirovski et al. 2012). Furthermore, studies in animal and human models showed that there is decreased ventilatory response to chronic hypercapnia implicating plasticity in the respiratory chemoreflex circuit (Burgraff et al. 2018; Schaefer 1963; Schaefer et al. 1963). Therefore, central respiratory chemoreception and associated  brain regions have various roles in the control of breathing, however despite these findings, there remains gaps in our understanding about the neurochemistry and neural circuit underlying these functions.

We have previously demonstrated that galanin is a mediator for adaptive changes in the respiratory chemoreflex circuit during chronic CO_2_ (Dereli et al. 2019). Galanin is an inducible peptidergic neuromodulator and galanin mRNA is expressed in many central respiratory chemoreceptor populations including the RTN, NTS, LC and lateral hypothalamic area (Nattie and Li 2002, 2010, 2012; Spirovski et al. 2012). Our results showed that following long term hypercapnia, galaninergic neurons in the RTN exhibited altered gene expression compared to room air control conditions and retained their central chemoreflex sensitivity as measured by c-Fos expression (Dereli et al. 2019). Moreover, microinjection of galanin into the preBötC attenuates ventilation and induces apnoea (Abbott et al. 2009), and we have demonstrated that galanin receptor 1 is expressed in the preBötC, serving as a substrate for these effects (Dereli et al. 2019). Taken together, there is strong evidence for the role of galanin in CO_2_ stimulated regulation of breathing.

The overall goal of this study is to identify and characterise neurons targeting the VRC. The preBötC, which forms the core of the respiratory rhythm generating circuitry within the VRC, is of particular interest. Due to its inherent rhythmic pacemaker-like properties, and role as a primary source of spontaneous inspiratory drive to premotor circuits, it emerges as a highly suitable target (Smith et al. 2009). Previous tracing studies demonstrated RTN projections to the preBötC in the mouse (Shi et al. 2021) and rat (Bochorishvili et al. 2012; Rosin et al. 2006). However, the neurochemistry and function of these projections still require further investigations. Although respiratory chemoresponsive areas containing galaninergic neuronal populations (namely the RTN, NTS, LC and lateral hypothalamic area) are recognised, along with  the presence of galanin receptors expressed in the VRC, whether galanin projections to the VRC facilitate respiration remains unknown. Given the existing knowledge gaps, our hypothesis posits that multiple galaninergic brain regions project to the VRC, including areas involved in CO_2_ stimulated breathing. Consequently, our study aims to achieve the following objectives: (1) Employ a mapping approach to identify all the brain projections to the VRC using a retrograde tracer centred into the preBötC. (2) Identify all galaninergic projections to the VRC. (3) Quantify the responsiveness of these VRC projections to acute hypercapnia through the assessment of c-Fos immunoreactivity.

Our findings collectively reveal that out of the 30 brain nuclei comprising 51 subnuclei that project to the VRC, only the RTN was recruited by the hypercapnia chemoreflex as indicated by c-Fos immunoreactivity. Furthermore, galaninergic inputs to the VRC originated from a limited number of nuclei out of which only the galaninergic RTN subset demonstrated significant neuronal activation in response to acute hypercapnia.

## Materials and methods

### Animals

All experimental procedures were approved by the Macquarie University Animal Ethics Committee (2018/024 and 2016/028) and carried out in accordance with the Australian Code of Practice for the Care and Use of Animals for Scientific Purposes and the National Health and Medical Research Council guidelines. Animals were group housed in conventional caging with 12:12 h, light:dark cycle at 23 °C with ad libitum access to standard chow and water.

### Retrograde tracer microinjections into the VRC

Anaesthesia was induced with 5% isoflurane in oxygen and maintained with 1–3% isoflurane. Once in the surgical plane of anaesthesia, mice were treated with prophylactic analgesia (Carprofen, 5 mg/kg, s.c.) and antibiotics (Cephazolin, 100 mg/kg, i.m.) and placed on a heating pad (~ 37 °C). Subcutaneous local anaesthetic (bupivacaine, 0.5%, 0.1 ml) was injected around the skull surface and an incision was made to expose the skull sutures. The overlying connective tissue was cleared, the skull flattened, and a small hole drilled, using a Neurostar stereotaxic robot, over the VRC (centered on the preBötC) ; 2.9 mm caudal to lambda and 1.3 mm lateral to midline.

**Fig. 1:**
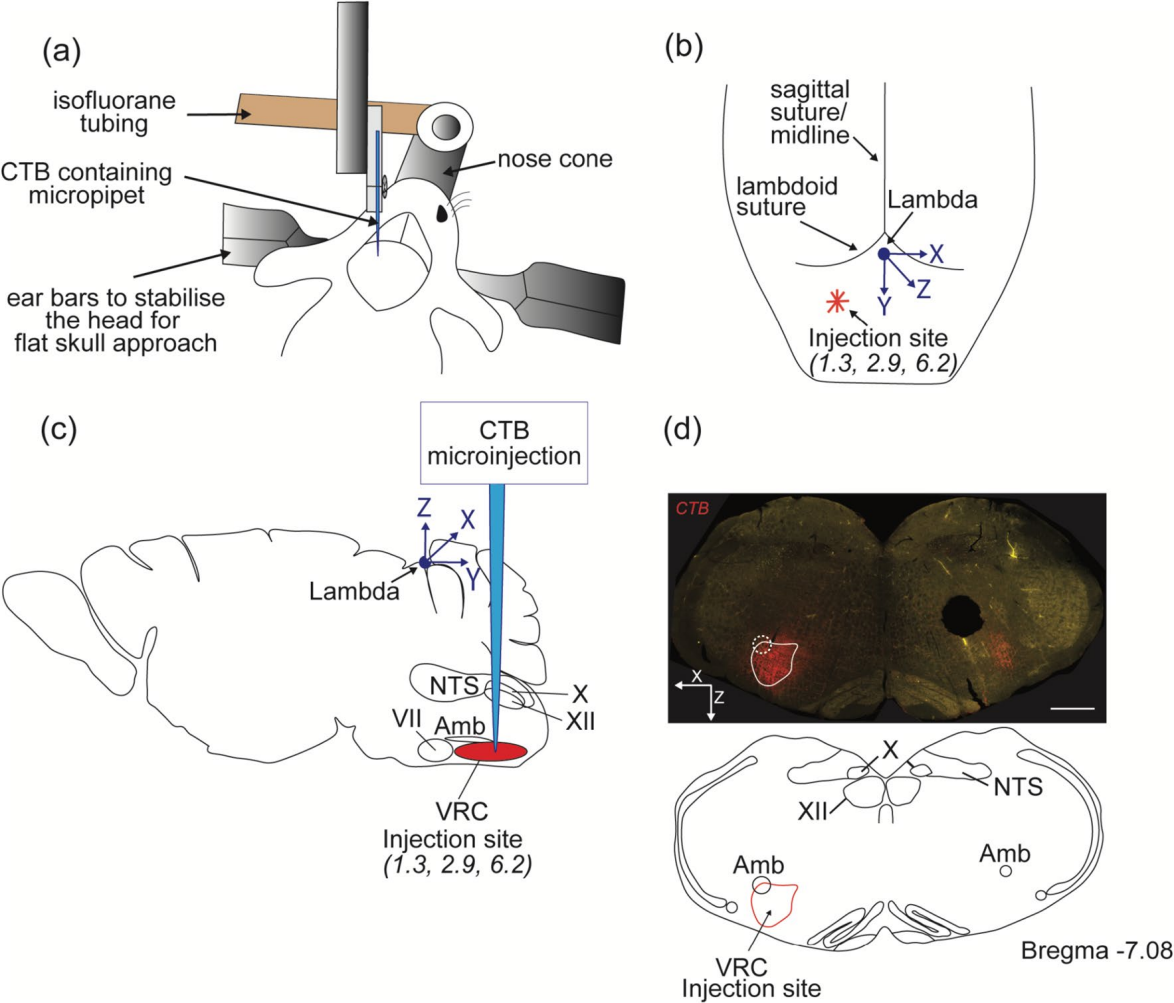
Schematic depiction of surgical approach and location of CTB injection site. **a** Mouse head mounted on the stereotaxic frame during CTB injection. The head of the mouse is positioned horizontal and symmetrical to the ear bars. There is continuous anaesthetic (isoflurane) fed to the animal through the nose cone to maintain a surgical plane of anaesthesia. **b** Schematic diagram of the frontal plane of the mouse skull indicating anatomical landmarks and stereotaxic coordinates of the injection site, which was 1.3 mm lateral to midline, 2.9 mm caudal to lambda and 6.2 mm ventral to the dorsal surface of the skull. **c** Sagittal schematic of the mouse brain indicating the location of CTB injection, the VRC, in relation to other brain areas and illustrating the anatomical trajectory of the micropipette reaching the *z*-coordinate. **d** A coronal brainstem image (−7.08 mm caudal to Bregma) showing an on-target CTB injection (red) in the location of the VRC, immediately ventral to the nucleus ambiguus (Amb). The scale bar is 500 μm

A 20 nl unilateral injection of cholera toxin B (CTB) was pneumatically injected over 3–5 min into the VRC using a glass capillary micropipette of diameter 20–40 μm at 6.2 mm deep to lambda (1%; List Biological, Campbell, CA) (Fig. 1). The pipette was left in place for 5 min before withdrawal. At the conclusion of injections, the incision was closed with silk sutures, isoflurane was discontinued, and once ambulatory, animals were returned to their home cage for 3–5 days to allow for CTB transport. Carprofen (5 mg/kg, s.c.) was administered once daily for pain relief.

### Acute hypercapnia challenge paradigm

After surgical recovery, mice were randomly assigned to either room air (RA) or acute hypercapnia (AH) challenge groups. A total of ten animals with on-target injection sites were used for analysis (5 RA vs 5 AH). On the final day of the experiment, AH mice were relocated to a quiet laboratory where they were exposed to 10% CO_2_/90% O_2_ in their home cages for 1 h, followed by 1 h in RA, prior to euthanasia. RA animals were treated identically, except that they breathed room air throughout the 2 h period. We used hyperoxic conditions (90% O_2_ as previously described; Guyenet et al. 2005; Mulkey et al. 2004, 2007) for the AH challenge to minimise input from peripheral oxygen sensors (Lahiri et al. 1987), ensuring that ventilatory responses to hypercapnia were mediated by central mechanisms.

At the end of the AH challenge, animals were deeply anaesthetised with sodium pentobarbital (> 150 mg/kg, ip) and transcardially perfused with heparinised 0.1 M phosphate buffered saline (PBS) followed by 4% paraformaldehyde (PFA, Sigma-Aldrich, NSW) in 0.1 M sodium phosphate buffer. Fixed brains were vibratome-sectioned coronally (30 μm) (Leica VT1200S, Leica, Germany) and stored in cryoprotectant solution (30% RNase-free sucrose, 30% ethylene glycol, 1% polyvinylpyrrolidone in 0.1 M sodium phosphate buffer, pH 7.4) at −20 °C.

### Non-radioactive chromogenic in situ hybridisation combined with immunohistochemistry

A template cDNA library was generated by reverse transcription of total RNA isolated from mouse brain tissue (Kumar et al. 2012). In situ hybridisation (ISH) riboprobes were synthesised from cDNA which was amplified from the cDNA library using a scaled-up PCR reaction. The PCR primers used for preprogalanin (ppGal) were ggatccatttaggtgacactatagaagCACCGAGAGAGCCTTGATCCTG (with SP6 RNA polymerase promoter regions attached at the 5′ end, in lowercase) and gaattctaatacgactcactatagggagaACGATTGGCTTGAGGAGTTGG (with T7 RNA polymerase promoter regions attached at the 5′ end, in lowercase). Following purification of the amplified cDNA template (Qiaquick gel extraction kit, Qiagen), riboprobes were prepared by in vitro transcription incorporating digoxigenin‐11‐UTP (Roche Applied Science, Mannheim, Germany) using the AmpliScribe T7 FLASH transcription kit (Epicentre Biotechnologies, Madison, WI, US) for antisense and SP6 RiboMAX large‐scale RNA production system (Promega, Madison, WI, US) for sense (control).

For digoxigenin (DIG) labelled ISH, free floating brain sections (1:6 (forebrain) & 1:3 series (brainstem)) were assayed as described previously (Spirovski et al. 2012; Kumar et al. 2012). Briefly, tissue sections were incubated with DIG-incorporated riboprobes (500–1000 ng/μl) overnight at 58 °C to allow for RNA hybridisation to occur. Sections were then rinsed through a series of decreasing salt concentrations and blocked prior to primary antibody incubation (24–48 h at 4 °C, antibody details are found in Table 1). Sections were rinsed with tris buffered saline (TBS) and incubated with fluorescently tagged secondary antibodies diluted in TBS containing 1% NHS and 0.1% Tween-20 at 4 °C overnight. ppGal mRNA expressing neurons were identified by detection of alkaline phosphatase activity following immersion of tissue sections in NBT/BCIP chromogenic substrate solution. Finally, sections were mounted and coverslipped with a mounting medium (Fluoroshield with DAPI or Fluoromount Aqueous Mounting Medium, Sigma, Australia).

**Table 1:** Primary, secondary, and tertiary antibodies used in this study

Antibody	Host (mono- vs polyclonal)	Working dilution	Company (catalog no)	RRID
Primary				
DIG	Sheep (pc)	1:1000	Roche (11093274910)	AB_2313640
CTB	Goat (pc)	1:3000	List Biological Laboratories (703)	AB_10013220
Phox2b	Guinea pig (pc)	1:1500	Gift from Professor Hideki Enomoto (Nagashimada et al. 2012)	n/a
c-Fos	Rabbit (pc)	1:4000	Santa Cruz (sc-253)	AB_2231996
TH	Mouse	1:1000	Millipore Sigma (MAB318)	AB_2927419
TH	Sheep	1:1000	Millipore (AB1542)	AB_2927420
Secondary				
α-Guinea pig Cy3	Donkey	1:400	Jackson ImmunoResearch (706-165-148)	
α-Rabbit 488	Donkey	1:400	Jackson ImmunoResearch (711-545-152)	
α-Goat Cy5	Donkey	1:400	Jackson ImmunoResearch (705-175-147)	
α-mouse AMCA	Donkey	1:200	Jackson ImmunoResearch (715-155-151)	
α-Mouse biotinylated	Donkey	1:1000	Jackson ImmunoResearch (715-065-151)	
α-Sheep Cy5	Donkey	1:400	Jackson ImmunoResearch (713-175-147)	
Tertiary				
Extravidin Cy3		1:500	Sigma-Aldrich (E4142-1ML)	

### Antibody characterisation

Sheep alkaline phosphatase-conjugated anti-DIG recognises the DIG-conjugated UTP incorporated into the riboprobes. Immunoblot assay demonstrated no cross reactivity of this antibody with any other known substance. Mouse brain tissue sections assayed in the absence of DIG-incorporated riboprobes lacked alkaline-phosphatase staining. The distribution of DIG-labelled ppGal riboprobes accorded with previous studies (Dereli et al. 2019).

**Fig. 2:**
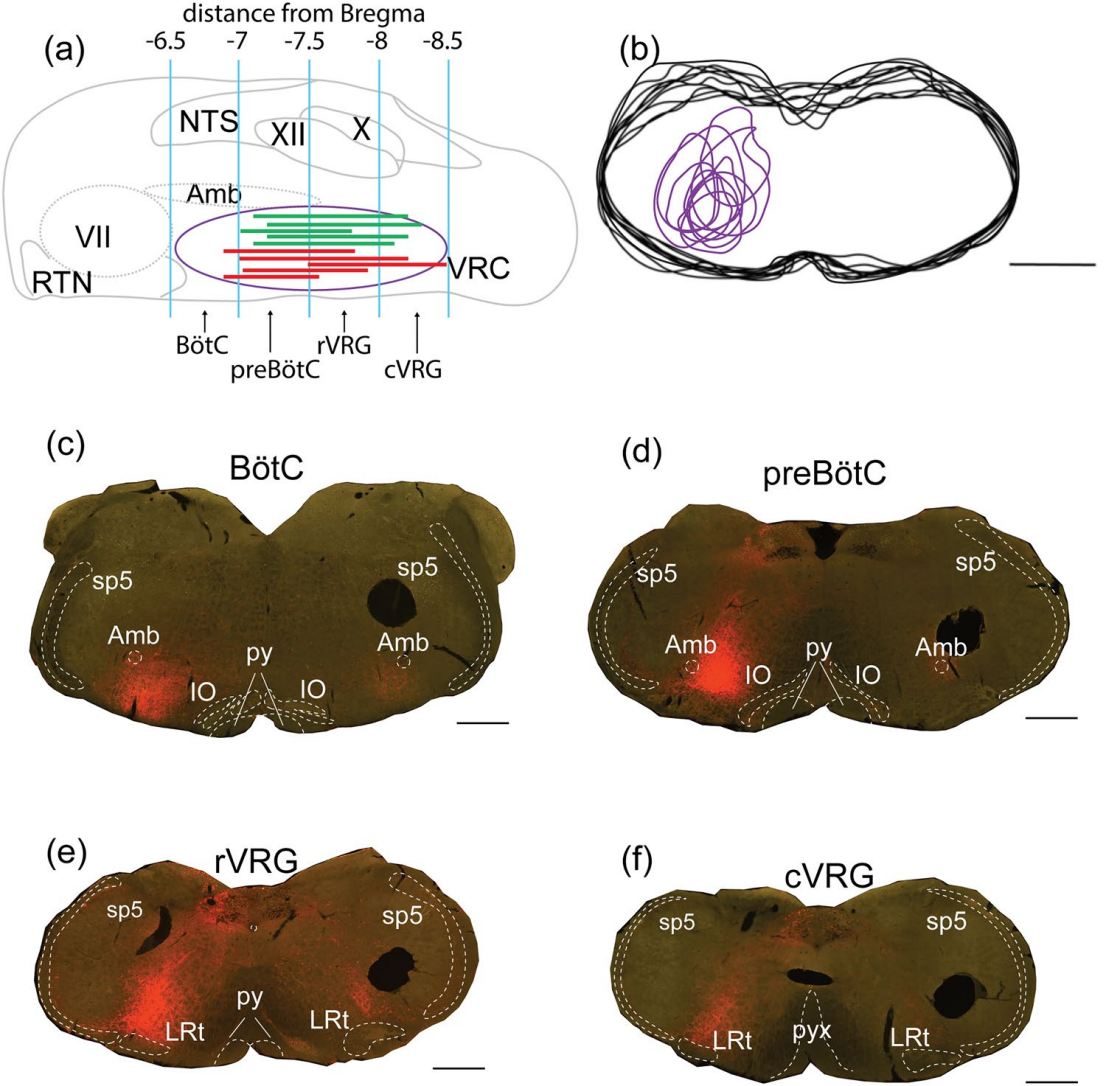
Verification of CTB injection site targeting the VRC. **a** Sagittal schematic of mouse brainstem illustrating rostrocaudal extent of the on-target injections (red and green lines) used for analysis. Each line in the VRC represents an animal: each green line represents a room air control animal; red lines represent animals that were exposed to 10% CO_2_, 1 h. **b** Schematic overlay of coronal sections from ten animals depicted in (**a**), showing dorsoventral and mediolateral extent of the on-target injection sites (purple). **c**–**f** Representative images from an animal with an on-target injection site at four different levels of the VRC showing CTB in red staining: **c** Bötzinger complex (BötC) at Bregma −6.84 mm, **d** preBötzinger complex (preBötC) at Bregma −7.32 mm, **e** rostral ventral respiratory group (rVRG) at Bregma −7.64 mm and **f** caudal ventral respiratory group (cVRG) at Bregma −8 mm. *RTN* retrotrapezoid nucleus; *VII* facial motor nucleus; *NTS* nucleus of the solitary tract ; *XII* hypoglossal nucleus; *X* dorsal motor nucleus of the vagus; *Amb* nucleus ambiguous; *LRt* lateral reticular nucleus; *pyx* pyramidal decussation; *sp5* spinal trigeminal tract; *IO* inferior olive. Scale bar is 500 µm for **b**, 1 mm for **c–f**

Rabbit anti-CTB targets the purified beta subunit of CTB and was previously characterised in other studies (Geerling et al. 2016). CTB is not endogenously produced in mammals. Anti-CTB staining was clearly distinguished on tissue sections at the level of the CTB injection site (Fig. 2c–f) and CTB labelling was absent in pilot studies.

Guinea-pig anti-Phox2b was a gift from Professor Hideki Enomoto (Kobe University, Kobe, Hyogo, Japan). Antigen specific immunolabeling was verified by co-expression with rabbit anti-Phox2b (gift from Professor Jean Francois Brunet, Ecole Normale Supérieure, Paris, France) and was consistent with previous reports of Phox2b expression in the adult mouse, as described previously by our group (Dereli et al. 2019). Specifically, rabbit anti-Phox2b antibody demonstrated labelling in catecholaminergic neurons and complete absence of immunoreactivity in Phox2b knockout mice (Lazarenko et al. 2009; Nagashimada et al. 2012).

Rabbit anti-c-Fos was previously characterised (Spirovski et al. 2012; Senthilkumaran et al. 2018; Fazekas et al. 2020). Western blot analyses showed that the antisera recognise Fos (62 kDa, Jurkat T lymphocyte cells), FosB, (20–60 kDa, human FosB transfected 293T lymphocyte cells), and Fra-1 and Fra-2 (23–43 kDa, human Fra-2 transfected 293T lymphocyte cells) proteins (Spirovski et al. 2012). After antibody titration, 1:4000 dilution gave specific optimum labelling with no background staining. c-Fos staining following acute hypercapnia challenge showed a staining pattern in respiratory nuclei, as previously described (Teppema et al. 1997).

Western blotting with mouse anti-tyrosine hydroxylase (TH) antibody confirmed that it specifically recognizes a 55–60 kDa protein in ventral tegmental area lysates (manufacturer's datasheet; Gumbs et al. 2019). Sheep anti-TH antibody was previously characterised by Baur et al. (2018). Western blot of rat brain corpus striatum homogenates show a single band of ∼62 kDa (manufacturer's datasheet; Haycock and Haycock 1991; Kansy et al. 2004). TH immunofluorescence for both antibodies was identical to previously described data (Lazarenko et al. 2009).

### Image processing, cell counts and analysis

Slides were scanned using either a Vectra Polaris Automated Quantitative Pathology Imaging System (Perkin Elmer, Waltham, MA, USA) or Zeiss Axioimager M2 (Carl Zeiss AG, Oberkochen, Germany) at the Mark Wainwright Analytical Centre, UNSW, Sydney. Neuronal profiles were plotted using StereoInvestigator software version 9 (Microbrightfield, USA). Tissue sections were aligned to the mouse stereotaxic brain atlas of Franklin and Paxinos (2007) (see Supplementary Information).

For qualitative analysis of neuronal profiles within a particular brain region, five sections per animal were screened. ppGal + neuronal distribution was graded as described previously (Bowman et al. 2013; Kuteeva et al. 2004; Verner et al. 2008): − (not expressed), + (scattered sparsely), +  + (expressed by < 1/3 of neurons in the area), +  +  + (expressed by > 1/3 of neurons in the area) (see Table 2, Supplementary Fig. 3).

**Table 2:** Relative abundance and CO_2_ responsiveness of neurons containing CTB, ppGal mRNA (*n* = 9-10)

Location	Nuclei and subnuclei	CTB +		CTB + /c-Fos +		CTB + /ppGal +
		Ipsil	Contral	RA	AH	
Cortical	Motor cortex:					
	Primary motor cortex (M1)	+	+	+	+	−
	Secondary motor cortex (M2)	+	+	+	+	−
	Insular cortex:					
	Dysgranular insular cortex (DI)	+	+	+	+	−
	Agranular insular cortex (AI)	+	+	+	+	−
	Somatosensory cortex:					
	Primary somatosensory cortex (S1)		+	+	+	−
	Secondary somatosensory cortex (S2)	+	+	+	+	−
	Secondary auditory cortex (Au)	+	+	+	+	−
	Temporal association cortex (TeA)	+	+	+	+	−
	Ectorhinal cortex (Ect)	+	+	+	+	−
	Perirhinal cortex (PRh)	+	+	+	+	−
Subcortical	Bed nucleus of stria terminalis (BNST)	+ +	−	+	+	+
	Amygdala:					
	Extended amygdala (EA)	+	−	+	−	−
	Central amygdaloid nucleus (CeA)	+ + +	+	+ +	+ +	+
	Medial amygdaloid nucleus (MeA)	+ + +	−	−	−	+
	Basolateral amygdaloid nucleus (BLA)	+ +	−	+	+	−
	Hypothalamus:					
	Anterior hypothalamic nucleus (AHN)	+	+	+	+	+
	Lateral preoptic area (LPA)	+ +	−	n/a	−	+
	Median preoptic nucleus (MPA)	+	−	+	+	+
	Dorsomedial hypothalamic nucleus (DMH)	+ + +	+	+	+	+
	Ventromedial hypothalamic nucleus (VMH)	+ +	+	+	+	+
	Paraventricular hypothalamic nucleus (PVN)	+ +	+	+	+	+
	Lateral hypothalamic area (LHA)	+ + +	+ +	+ +	+ +	+
	Thalamus:					
	Ventromedial thalamic nucleus (VM)†	+	+ + +	−	−	−
	Ventral posteromedial thalamic nucleus (VPM)†	+	+ + +	−	−	−
	Submedius thalamic nucleus (STh)†	+	+ +	+	+	−
	Reticular formation	+	−	−	n/a	−
	Interfascicular nucleus (IF)	+	+	−	n/a	−
	Zona incerta (ZI)	+	+	+	−	−
	Anterior pretectal nucleus (APT)	−	+ +	−	n/a	−
Midbrain	Periaqueductal gray (PAG):					
	Dorsomedial periaqueductal gray (DMPAG)	+ +	+	−	−	−
	Ventrolateral periaqueductal gray (VLPAG)	+ +	+	+	+	−
	Lateral periaqueductal gray (LPAG)	+ + +	+	+	+	−
	Intermediate white layer of superior colliculus (SC)	−	+ + +	−	n/a	−
Pons	Edinger-Westphal nucleus (EW)	+ + +	+ + +	−	n/a	−
	Lateral parabrachial nucleus (LPB)	+ +	+	+	+	−
	Medial parabrachial nucleus (MPB)	+ +	+	+	+	−
	Kölliker-Fuse (KF)	+ + +	+	+	+	−
	Barrington’s nucleus (Bar)	+	−	−	−	−
	Pontine reticular nucleus (PnO)	+	+	−	−	−
	Raphe magnus (RMg)	+	+	−	+	−
	Subcoeruleus nucleus (SubC)	+ +	+	+	+	−
Medulla	A5 region	+ +	+	−	−	−
	Intermediate reticular nucleus (IRt)	+	+	−	−	−
	Raphe pallidus (RPa)	+	+	+	+ +	−
	Raphe magnus (RMg)	+	+	−	+	−
	Retrotrapezoid nucleus (RTN)	+ +	+	+	+ +	+ + +
	Ventral respiratory column (VRC)	n/a	+ +	−	+	−
	Cuneate nucleus (Cu)	+ +	−	−	−	−
	Nucleus of the solitary tract (NTS)	+ + +	+	−	+	+ +
	Area postrema (AP)	+ + +	+	−	+	−
	Spinal trigeminal nucleus (Sp5)	+ +	−	−	−	−

All anatomical areas listed are according to the mouse stereotaxic atlas (Franklin and Paxinos 2007). For each animal, five sections per area were analysed. Refer to Supplementary Fig. 3 for the qualitative analysis scale used to grade cellular expression

− undetectable; + scattered sparsely; +  + expressed by 1/3 of the neurons in the area; +  +  + expressed by > 1/3 of the neurons in the area; *n/a* not available; *AH* acute hypercapnia challenge; *RA* room air

^†^Processes

For quantitative analysis, only neurons with DAPI-stained nuclei were considered for counting. Since few CTB + neurons were detected on the contralateral side (<1/5), counts were made only from the ipsilateral side. Phox2b and TH were used to assist identification of defined neuronal populations, i.e. the RTN, NTS, LPB and KF. CTB, Phox2b, and ppGal mRNA expressing cells were counted. c-Fos + cells were only counted if they co-localised with another neuronal marker, such as CTB or ppGal. Cellular profiles were manually counted for all analyses except for the ppGal chromogenic mRNA stain in the forebrain which was counted semi-automatically using the ‘positive cell detection’ function in Qupath software (Bankhead et al. 2017). Total cell counts were obtained following Abercrombie correction (Abercrombie 1946): for 30 μm thick sections, an average nuclear width of 7.2 ± 0.2 μm and average section thickness of 29.7 ± 1 μm was measured from 30 cells per section, in 10 sections from each of 10 animals. All values are given ± SEM values and comparisons were initially performed using two-way ANOVA. Where required, post-hoc analysis was conducted using Sidak’s methods on GraphPad Prism. Representative images were first imported into Fiji (RRID:SCR_002285) as TIFF files for brightness/contrast adjustment to increase the clarity and to reflect true rendering. Images were not otherwise altered. TIFF images were then imported into CorelDraw Graphics Suite X7 (64-bit) or Adobe Illustrator CC (2019) for final presentation.

## Results

### Verification of CTB injection site targeting the VRC

Figure 2a, b summarises the locations of injection sites from experiments included in this study. We successfully targeted the VRC; CTB deposits were centred at the preBötC and rVRG in 9/10 injections (Bregma −7.2 to −7.7 mm) and rVRG and cVRG in 1/10 injections (Bregma −6.9 to −8.5 mm) (Fig. 2a). Figure 2b illustrates the mediolateral and dorsoventral extent of the 10 CTB injections used for quantitative analysis. Figure 2c–f shows representative coronal images from an animal with an on-target injection site at four different compartments of the VRC.

### Brain nuclei that project to the VRC

Qualitative analysis (Table 2) identified 30 brain nuclei and 51 subnuclei that project to the VRC which were later quantified in Fig. 6. The majority of CTB labelled neurons were located ipsilateral to injection sites (See Supplementary Fig. 4). In the neocortex, CTB + neurons were identified in the motor, insular, somatosensory, auditory, temporal association and rhinal cortices. Brainstem regions that contained CTB + cell bodies included the PAG, superior colliculus (SC), KF, LPB, Edinger–Westphal nucleus, Barrington’s nucleus, subcoeruleus nucleus (SubC), RTN, NTS, cuneate nucleus, trigeminal nucleus, area postrema, reticular nucleus, and VRC (Table 2).

**Fig. 3:**
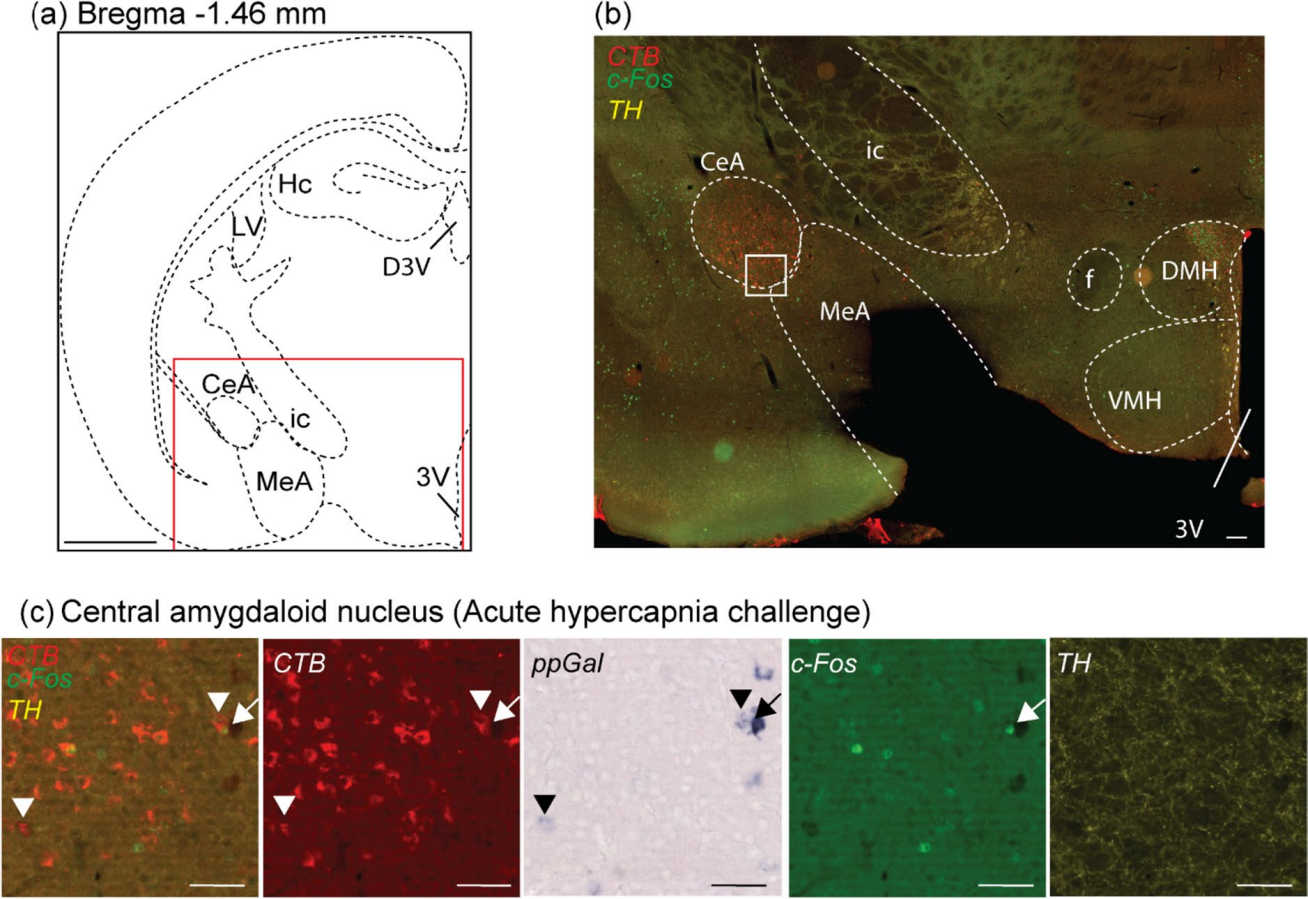
A subpopulation of central amygdaloid nucleus (CeA) neurons projected to the VRC, are galaninergic, but not c-Fos + . **a** Schematic brain hemisection corresponding to Bregma −1.46 mm. The inset in (**a**) is enlarged in (**b**), showing the relative locations of the CeA and MeA. **c** is a high magnification image of the inset in (**b**), with CTB (red), ppGal (brightfield), c-Fos (green) and TH (yellow) labelling after acute hypercapnia challenge. Arrowheads point to VRC-projecting neurons that are ppGal + and are not c-Fos + . Arrow points to a ppGal + neuron that does not project to the VRC and is c-Fos +. Scale bars are 1 mm in (**a**), 100 µm in (**b**) and 50 µm in (**c**). Schematic diagram (**a**) is adapted from Franklin and Paxinos (2007). *ic* internal capsule; *VMH* ventromedial hypothalamic nucleus; *DMH* dorsomedial hypothalamic nucleus; *f* fornix; *3V* third ventricle; *Hc* hippocampus; *ic* internal capsule; *LV* lateral ventricle; *D3V* dorsal 3rd ventricle

**Fig. 4:**
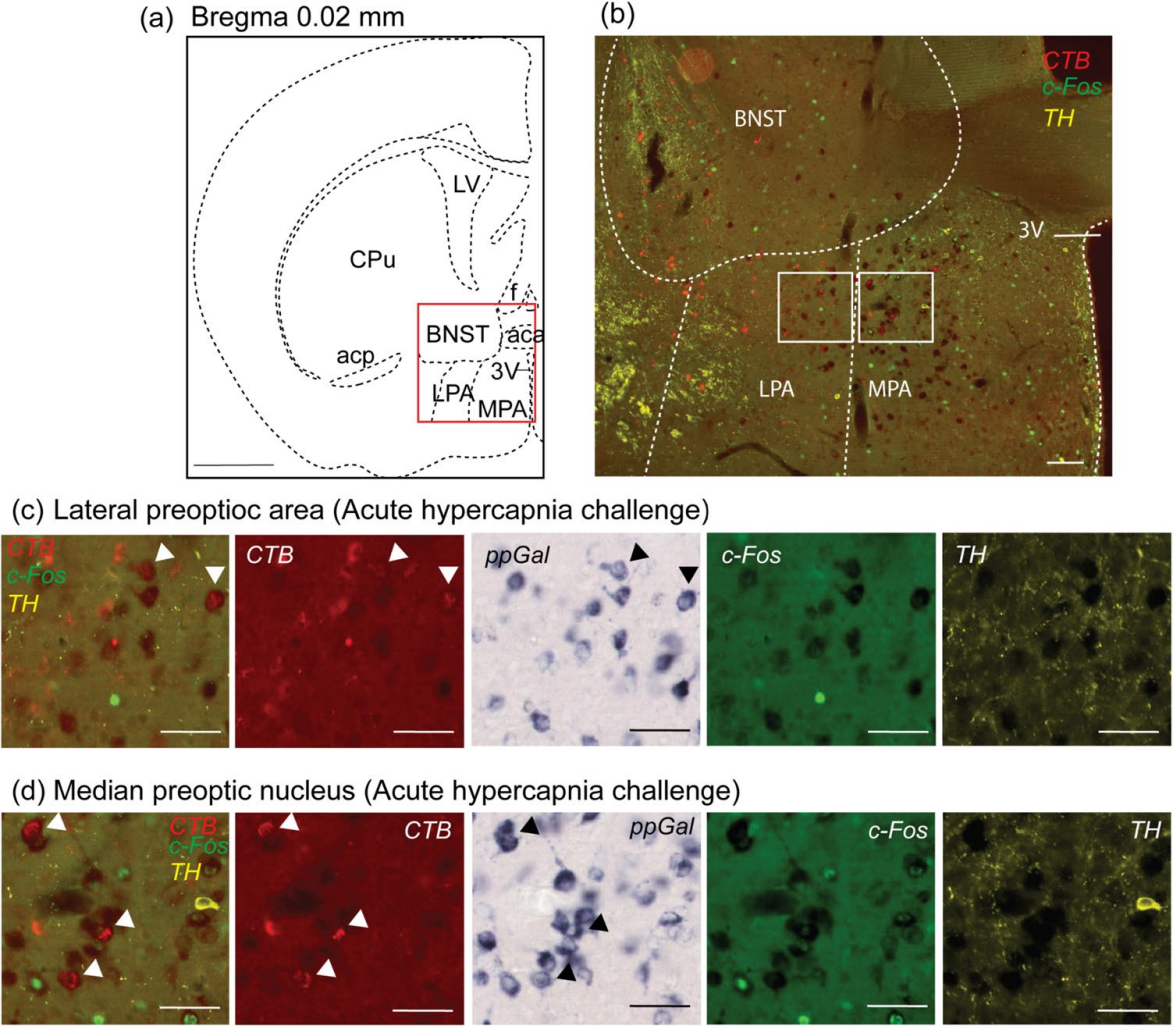
A subpopulation of VRC-projecting lateral preoptic area (LPA) and median preoptic nucleus (MPA) neurons are galaninergic but not responsive to acute hypercapnia challenge. **a** Schematic of a brain section corresponding to Bregma 0.02 mm. The red box in (**a**) is depicted in (**b**) which is a representative low resolution photomicrograph of the LPA and MPA containing ppGal, CTB, c-Fos and TH labelling and major landmarks. Higher magnification of the insets in (**b**) are presented in panels (**c**) and (**d**) with CTB (red), ppGal (brightfield), c-Fos (green) and TH (yellow) labelling after acute hypercapnia challenge. Arrowheads point to examples of ppGal + neurons that project to the VRC. None of the projecting ppGal + neurons were activated following acute hypercapnia. Scale bars are 1 mm in (**a**), 100 µm in (**b**) and 50 µm in (**c**) and (**d**). Schematic diagram (**a**) is adapted from Franklin and Paxinos (2007). *BNST* bed nucleus of stria terminalis; *3V* third ventricle; *CPu* caudate putamen; *acp* anterior commissure posterior nerve; *aca* anterior commissure anterior part; *LV* lateral ventricle; *f* fornix

**Fig. 5:**
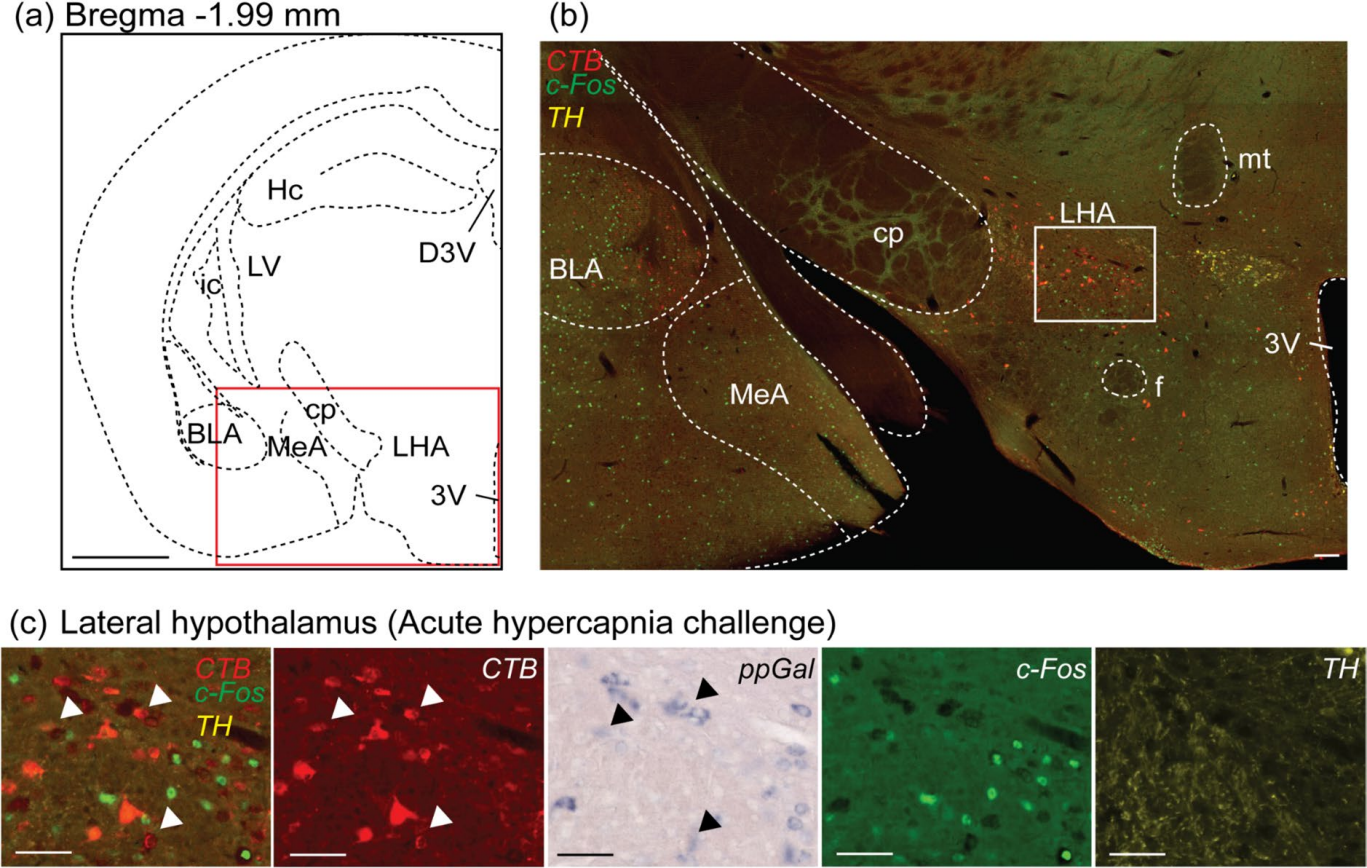
A subpopulation of VRC projecting lateral hypothalamus (LHA) neurons are galaninergic but not responsive to acute hypercapnia challenge when compared to control conditions. **a** Schematic of a brain section corresponding to Bregma −1.99 mm. The red box in (**a**) is the area depicted in (**b**) showing the relative location of the LHA. (**c**) is a high magnification image of the inset in (**b**), with CTB (red), ppGal (brightfield), c-Fos (green) and TH (yellow) labelling after acute hypercapnia challenge. Arrowheads point to examples of ppGal + neurons projecting to the VRC. None of these ppGal + neurons were activated following acute hypercapnia, although there were many c-Fos + neurons in the area. Scale bars are 1 mm in (**a**), 100 µm in (**b**) and 50 µm in (**c**). Schematic diagram (**a**) is adapted from Franklin and Paxinos (2007). *BLA* basolateral amygdaloid nucleus; *MeA* medial amygdaloid nucleus; *f* fornix; *mt* mammillothalamic tract; *Hc* hippocampus; *cp* cerebral peduncle; *ic* internal capsule; *LV* lateral ventricle; *D3V* dorsal 3rd ventricle

**Fig. 6:**
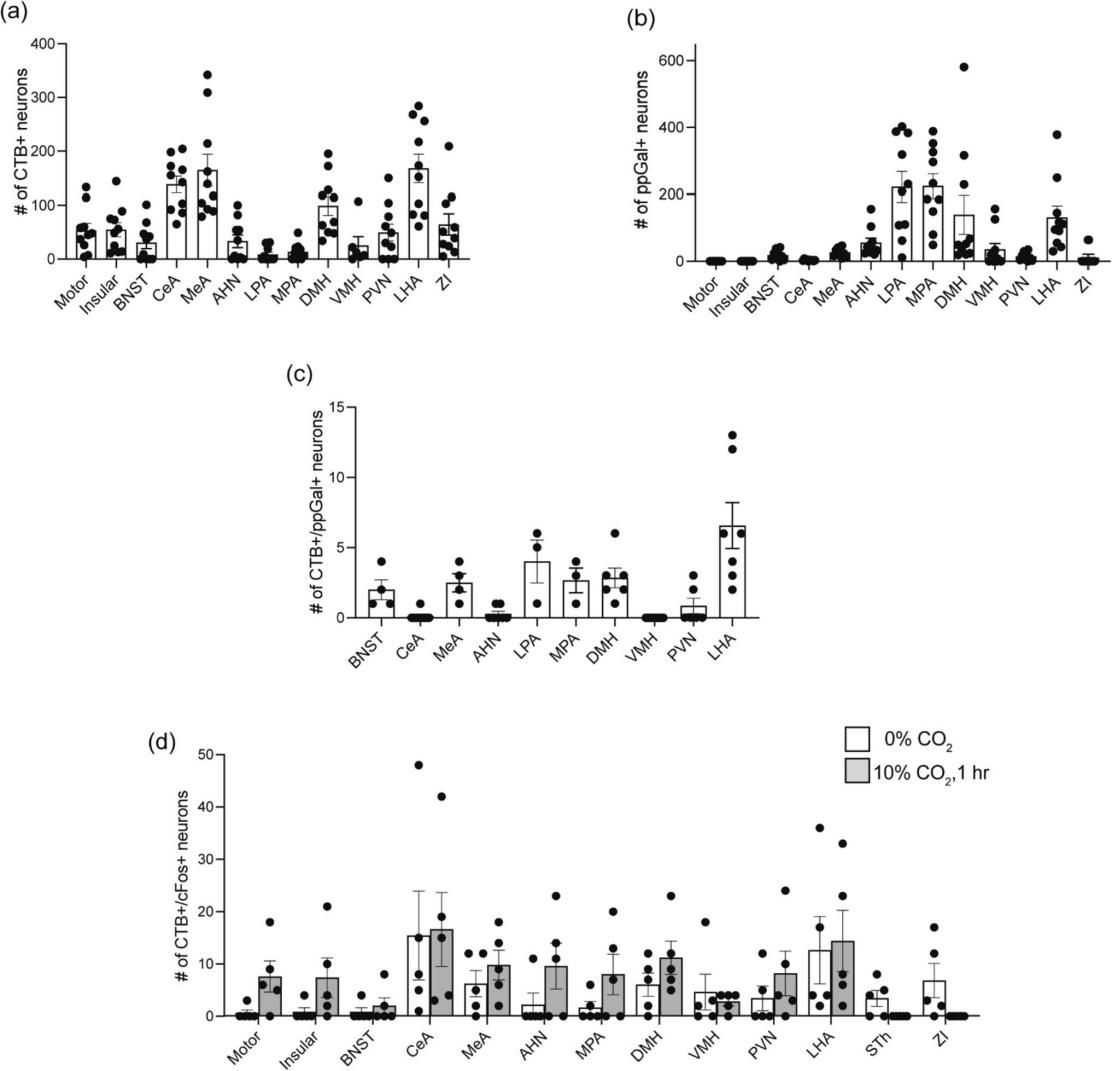
Neural populations in the forebrain that project to the VRC, including a galaninergic subset, are not activated by acute hypercapnia challenge. **a** Total number of VRC-projecting (CTB +) neurons across different neural populations in the forebrain. **b** Total number of ppGal + neurons across different neural populations in the forebrain. **c** Number of VRC projecting (CTB +) neurons that are double labelled with ppGal. **d** Comparison of the VRC-projecting and activated (CTB + , c-Fos + double labelled) neuronal counts between the room air and acute hypercapnia groups [two-way ANOVA, Sidak’s post-hoc test: *p* > 0.05 for all multiple comparisons, not significant, (*n* = 5)]. All values are given ± SEM values. No less than six sections per area per animal was analysed

In the amygdala, projecting neurons were identified in the extended amygdala (EA), central (CeA), medial (MeA) (Fig. 3) and basolateral amygdaloid nucleus (BLA). In the hypothalamus, VRC-projecting subnuclei were identified in the preoptic areas (Fig. 4), DMH, ventromedial hypothalamic nucleus (VMH), paraventricular hypothalamic nucleus (PVN) and lateral hypothalamic area (LHA) (Fig. 5). Quantitatively, the forebrain population with the most abundant CTB + neurons was LHA (168 ± 27, *n* = 10), followed by MeA (165 ± 30, *n* = 10), CeA (138 ± 15, *n* = 10) and DMH (99 ± 17, *n* = 10) (Fig. 6a).

**Fig. 7:**
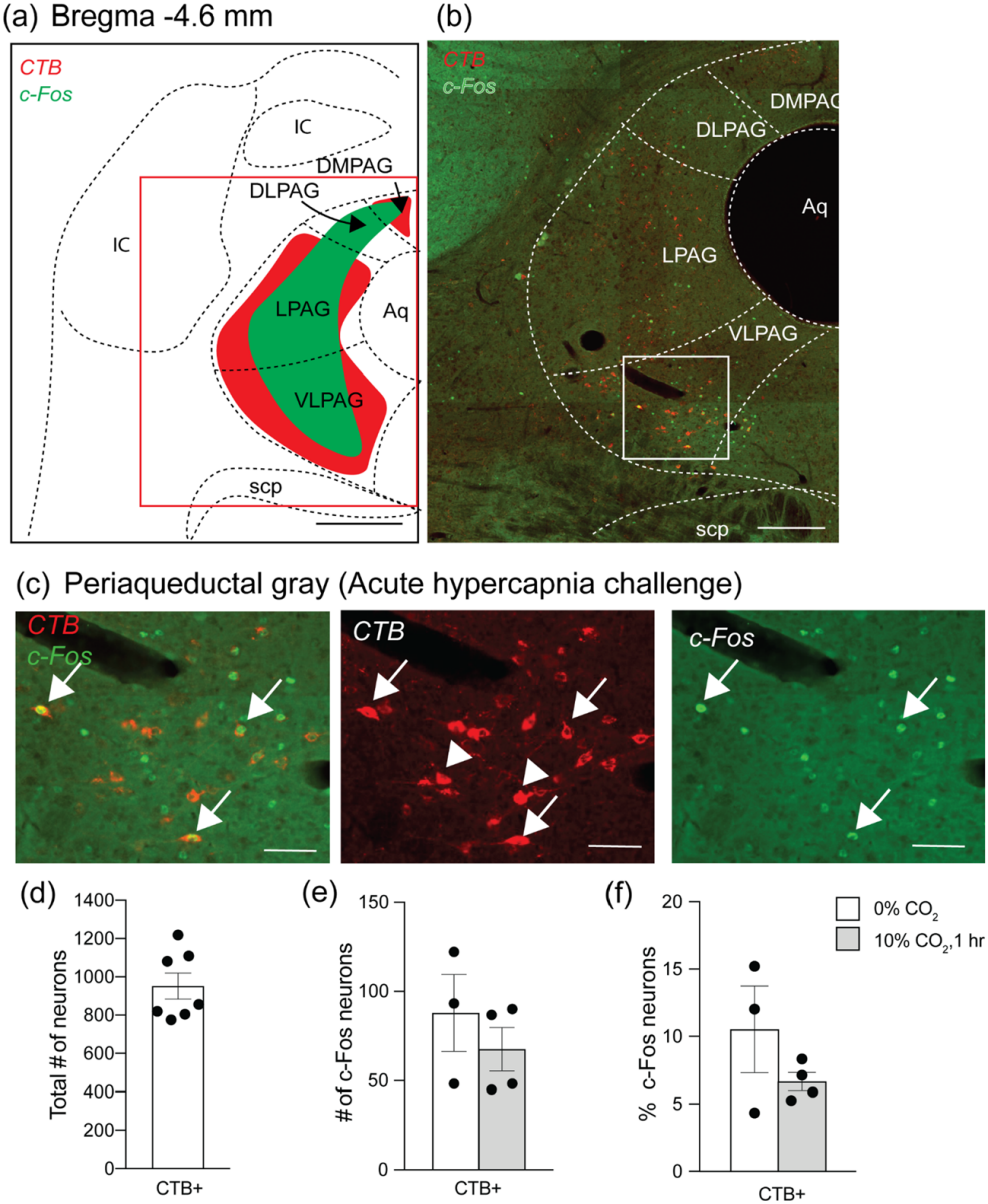
The periaqueductal gray (PAG) neurons project to the VRC but are not activated by acute hypercapnia challenge when compared to control conditions. **a** Schematic of a brain section corresponding to Bregma -4.6 mm. The red box in (**a**) is the area depicted in (**b**) which shows the relative location of the PAG. VRC projecting neurons (red) were mainly distributed in dorsomedial, lateral and ventrolateral subnuclei of the PAG (DMPAG, LPAG and VLPAG respectively). (**c**) is the higher magnification image of the inset in (**b**) with CTB (red) and c-Fos (green) labelling after AH challenge. Arrowheads point to examples of PAG neurons projecting to the VRC that are not c-Fos + and arrows point to VRC projecting PAG neurons that are c-Fos + . **d** Total VRC projecting PAG neurons. **e** Total projecting PAG neurons that are activated (c-Fos +) following acute hypercapnia challenge. **f** Percentage projecting PAG neurons that are activated following acute hypercapnia challenge (*n* = 3–4) (two-way ANOVA: *p* < 0.05). (**a**) is adapted from Franklin and Paxinos (2007). Scale bars are 500 μm in (**a**) 200 μm in (**b**) and 50 μm in (**c**). *IC* inferior colliculus; *scp* superior cerebellar peduncle; *Aq* aqueduct

There were CTB + neurons throughout the PAG, including the dorsomedial (DMPAG), lateral (LPAG) and ventrolateral compartments (VLPAG) (Fig. 7a, b). Counts between Bregma levels -4.72 and -4.48 showed an average of 951 ± 68 (*n* = 7) VRC projecting neurons within these compartments (Fig. 7d).

**Fig. 8:**
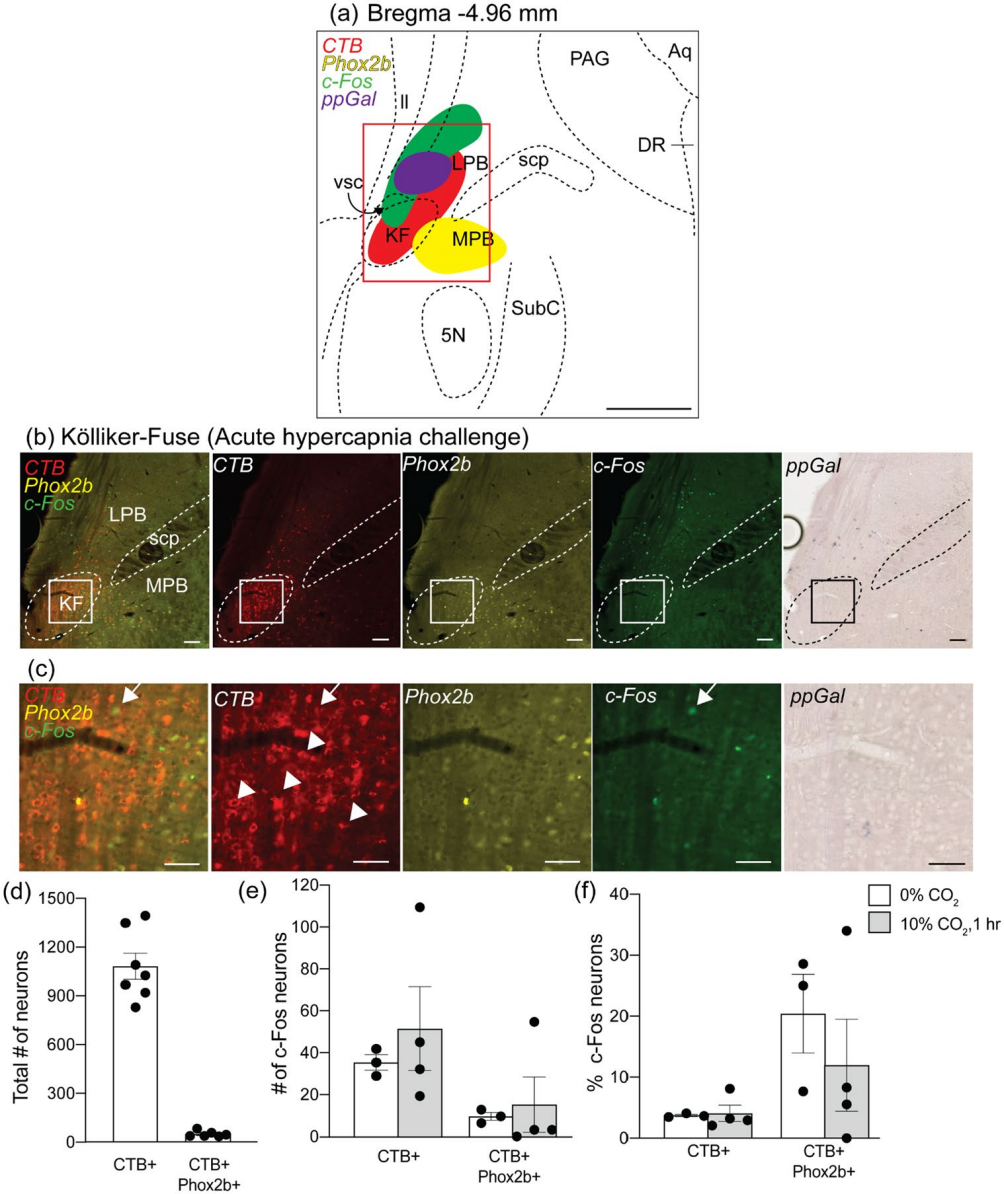
The Kölliker–Fuse (KF) neurons project to the VRC but were not ppGal + or activated by acute hypercapnia challenge when compared to control conditions. **a** Schematic of a brainstem section corresponding to Bregma −4.96 mm depicting the observed pattern of neurons expressing Phox2b (yellow), ppGal mRNA (brightfield), CTB (red) and c-Fos (green). The red box in (**a**) is enlarged in (**b**) which represents the KF region from an animal following acute hypercapnia challenge. The inset in (**b**) is magnified in (**c**) and corresponds to the core of the KF dorsoventrally. Arrowheads point to examples of KF neurons that project to the VRC and are not c-Fos + whereas arrows point to VRC projecting KF neurons that are c-Fos + . None of the CTB + neurons were galaninergic and very few were activated. **d** The total number of VRC projecting and VRC projecting Phox2b + KF neurons within the ipsilateral brain. **e** The total number of VRC projecting and VRC projecting Phox2b + KF neurons that are activated (c-Fos +) following acute hypercapnia challenge within the ipsilateral brain. **f** Percentage of VRC projecting and VRC projecting Phox2b + KF neurons that are activated following acute hypercapnia challenge. (*n* = 3–4) (two-way ANOVA: *p* > 0.05). (**a**) is adapted from Franklin and Paxinos (2007). Scale bars are 500 µm in (**a**), 100 µm in (**b**) and (**c**), 50 µm in (**d**) and (**e**). *MPB* medial parabrachial nucleus; *scp* superior cerebellar peduncle; *vsc* ventral spinocerebellar tract; *ll* lateral lemniscus; *PAG* periaqueductal gray; *DR* dorsal raphe; *5N* motor trigeminal nucleus; *SubC* subcoeruleus nucleus; *Aq* aqueduct

**Fig. 9:**
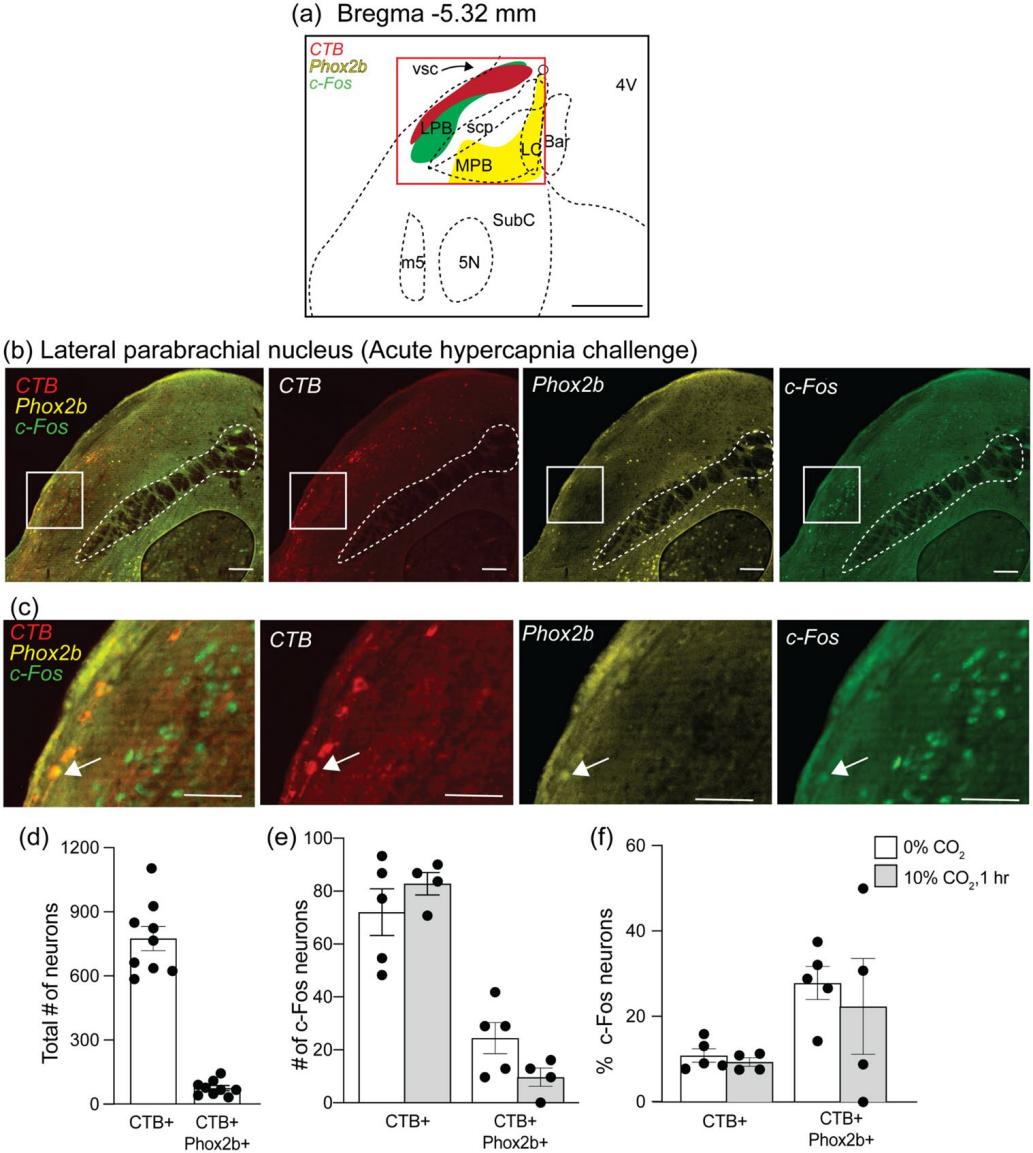
The lateral parabrachial nucleus (LPB) contains neurons that project to the VRC but were not ppGal + or activated by acute hypercapnia challenge when compared to control conditions. **a** Schematic of a brain section corresponding to Bregma −5.32 mm. Distinct populations of neurons expressing Phox2b (yellow), CTB (red) and c-Fos (green) were observed. The red box in (**a**) is the area depicted in (**b**) which is a representative image following acute hypercapnia challenge. (**c**) is a high magnification image of the inset in (**b**). The arrow points to an example of Phox2b + LPB neuron that projects to the VRC and is c-Fos + . **d** Total VRC projecting and VRC projecting Phox2b + LPB neurons within the ipsilateral brain. **e** Total VRC projecting and VRC projecting Phox2b + LPB neurons that are activated (c-Fos +) following acute hypercapnia challenge within the ipsilateral brain. **f** Percentage VRC projecting and VRC projecting Phox2b + LPB neurons that are activated following acute hypercapnia challenge (*n* = 4–5) (two-way ANOVA: *p* > 0.05). **a** is adapted from Franklin and Paxinos (2007). Scale bars are 500 μm in (**a**), 100 μm in (**b**), 50 μm in (**c**). *MPB* medial parabrachial nucleus; *scp* superior cerebellar peduncle; *vsc* ventral spinocerebellar tract; *m5* motor root of the trigeminal nerve; *5N* motor trigeminal nucleus; *SubC* subcoeruleus nucleus; *4V* fourth ventricle; *LC* locus coeruleus; *Bar* Barrington’s nucleus

**Fig. 10:**
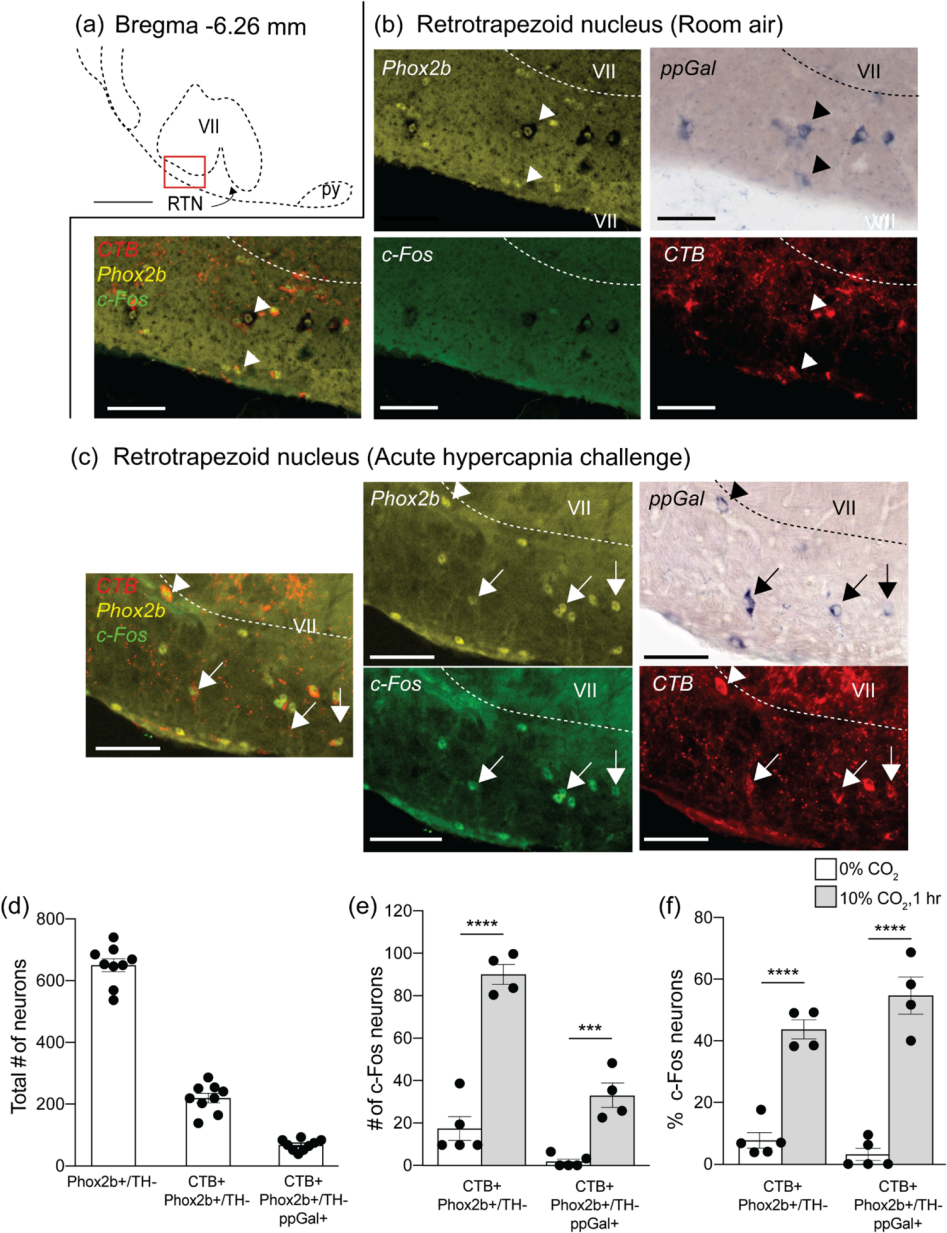
The retrotrapezoid nucleus **(**RTN) contains ppGal + VRC projecting neurons that are activated by acute hypercapnia challenge. **a** Schematic of the ipsilateral side of a brainstem section corresponding to Bregma −6.26 mm. The red box in (**a**) is the area depicted in **b** and **c** which are representative images of the RTN region immunostained for Phox2b (yellow), ppGal mRNA (brightfield), CTB (red) and c-Fos (green) following **b** room air and **c** acute hypercapnia challenge. Arrowheads point to examples of galaninergic RTN neurons that project to the VRC but are not c-Fos + . Arrows point to ppGal + VRC projecting neurons that are c-Fos + . **d** Total RTN (Phox2b + /TH−) neurons, VRC projecting RTN (CTB +) neurons and VRC projecting galaninergic RTN neurons within the ipsilateral brainstem. **e** Total VRC projecting RTN neurons and galaninergic RTN neurons that are activated (c-Fos +) following acute hypercapnia challenge within the ipsilateral brainstem. **f** The percentage of VRC projecting RTN neurons and galaninergic RTN neurons that are activated following acute hypercapnia challenge (*n* = 4–5) (two-way ANOVA: *p* < 0.05, Sidak’s post-hoc test: ***p < 0.001, ****p < 0.0001). (**a**) is adapted from Franklin and Paxinos (2007). Scale bars are 500 µm in (**a**), 50 µm in (**b**) and (**c**). *VII* facial motor nucleus; *py* pyramid

In the pons, the LPB and KF regions contained CTB + neurons throughout the entire rostrocaudal extent of each nucleus (Figs. 9a and 10a). In the KF (Bregma −5.08 and −4.84), an average of 1082 ± 80.6 neurons (*n* = 7) were CTB + of which 5.9% was Phox2b + (50 ± 8 total cell counts, *n* = 7) (Fig. 8d). This represented 23.9% of all Phox2b + neurons in the KF area (346 ± 59 total cell counts, *n* = 7). In the LPB (Bregma levels −5.44 and −5.2), there was an average of 775 ± 56 (*n* = 9) CTB + neurons out of which 10.5% were Phox2b + neurons (75 ± 1 total cell counts, *n* = 9) (Fig. 9d).

RTN neurons are characterised to be Phox2b + (a transcription factor) and TH-, therefore counts were made from Phox2b + /TH- neurons in the parafacial region. 33.5% of RTN neurons (219 ± 15 total cell counts, *n* = 9) were immunoreactive for CTB on the ipsilateral side (Fig. 10d).

**Fig. 11:**
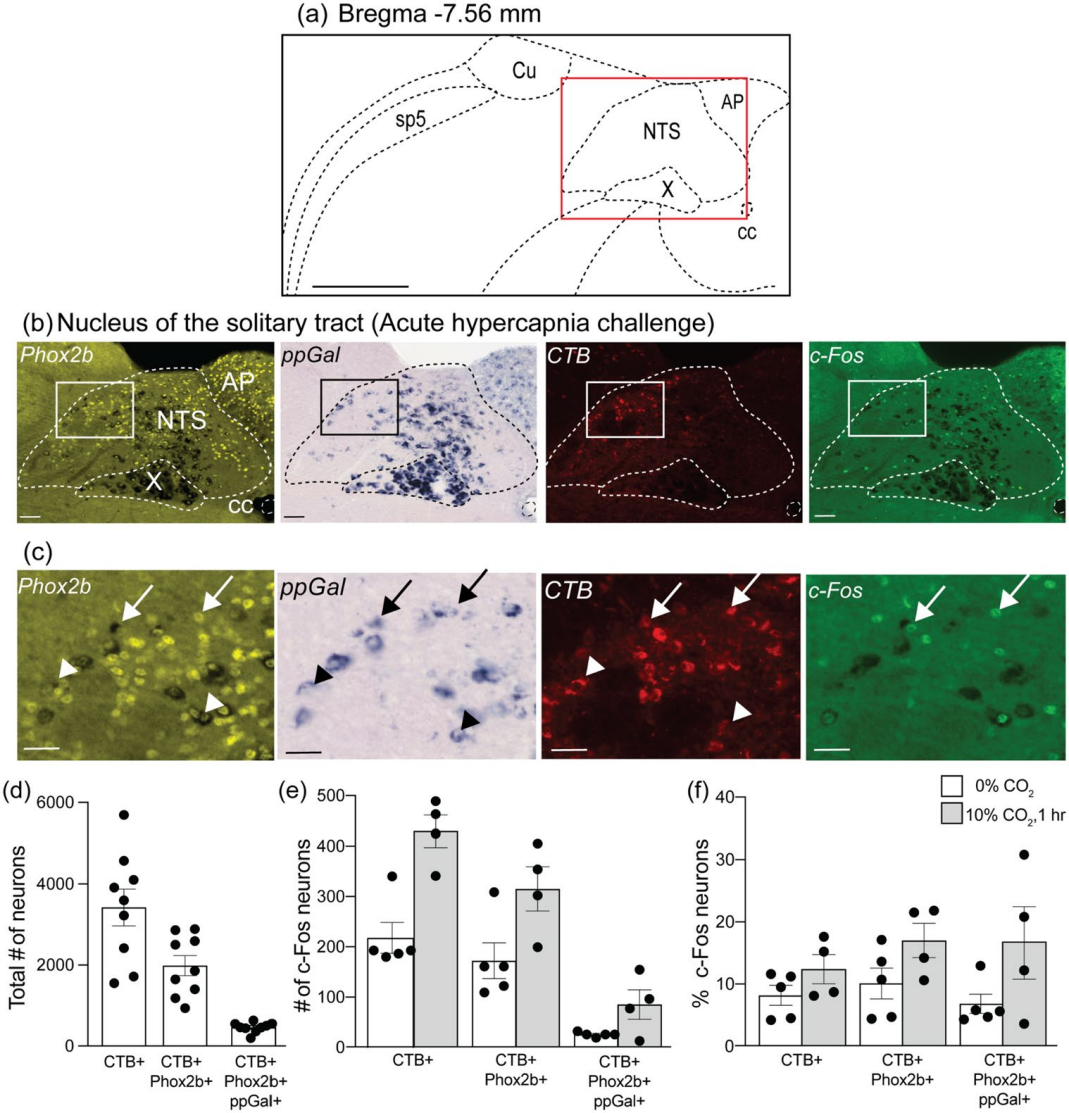
The nucleus of the solitary tract (NTS) contains galaninergic neurons projecting to the VRC (ipsilateral to the injection site); however these are not activated by acute hypercapnia challenge when compared to control conditions. **a** Coronal brainstem schematic corresponding to Bregma −7.56 mm. The red box in (**a**) is the area depicted in (**b**) which is a representative low resolution photomicrograph of the caudal NTS containing Phox2b (yellow), ppGal (brightfield), CTB (red) and c-Fos (green) staining and major landmarks. Higher magnification of the insets in (**b**) are presented in panel (**c**). Arrowheads point to examples of NTS neurons that project to the VRC and are ppGal + but not c-Fos + and arrows point to examples of ppGal + NTS neurons that project to the VRC and are c-Fos + . **d** Total number of ipsilateral cNTS neurons that project to the VRC (CTB +) and the proportion that are also Phox2b + or ppGal + . **e** Total VRC projecting, Phox2b + and galaninergic cNTS neurons that are activated (c-Fos +) following acute hypercapnia challenge within the ipsilateral hemisphere. **f** Percentage of VRC projecting, Phox2b + and galaninergic cNTS neurons that are activated following acute hypercapnia challenge. (*n* = 4–5) (two-way ANOVA: *p* > 0.05). (**a**) is adapted from Franklin and Paxinos (2007). Scale bars are 500 µm in (**a**), 50 µm in (**b**) and 25 µm in (**c**). *AP* area postrema; *X* dorsal motor nucleus of the vagus; *sp5* spinal trigeminal tract; *Cu* cuneate nucleus; *cc* central canal

In the NTS, CTB immunoreactivity was mainly observed in the caudal division (cNTS). In the rostral NTS (rNTS), defined by disappearance of the area postrema, no CTB + neurons were detected. Therefore, quantitative analysis was only performed for the cNTS (Bregma levels -8.04 and -6.84). An average of 3420 ± 451 neurons (*n* = 9) were CTB + and 59.9% of these neurons were Phox2b + (1986 ± 248 total cell counts, *n* = 9) (Fig. 11d).

### Multiple VRC projecting neuronal populations in the forebrain express ppGal mRNA

The forebrain regions that express ppGal mRNA were identified (Table 2) and quantified (Fig. 6b). ppGal ISH product was observed in motor and insular cortices, bed nucleus of stria terminalis (BNST), CeA, MeA, anterior hypothalamic nucleus (AHN), lateral (LPA) and medial (MPA) preoptic areas, DMH, VMH, PVN, LHA and zona incerta (ZI). Amongst these, the region that contained the highest mean count of ppGal + neurons, was MPA (224 ± 36, *n* = 8), followed by LPA (222 ± 46, *n* = 8), DMH (139 ± 59, *n* = 10) and LHA (132 ± 34, *n* = 10).

Two regions (hypothalamus, amygdala) and 10 associated subnuclei (BNST, CeA, MeA, AHN, LPA, MPA, DMH, VMH, PVN and LHA) contained double labelled neurons that project to the VRC and express ppGal (Fig. 6c). LPA had the highest proportion of VRC-projecting neurons that express ppGal (3.6%, 4 ± 2.5 total cell counts, *n* = 5), followed by the LHA (3.3%, 6.6 ± 4.3 total cell counts, *n* = 5). The PVN and the VMH had distinct CTB + and ppGal + neuronal populations, but no ppGal + /CTB + double labelled neurons were detected.

In the brainstem, only the RTN (Fig. 10a–c) and NTS (Fig. 11a–c) contained VRC projecting neurons that also express ppGal mRNA (CTB + /ppGal +). In the RTN, 31.7% (68 ± 6 total cell counts, *n* = 9, Fig. 10d) of CTB + neurons were ppGal + , and in the NTS, 14.8% (462 ± 42 total cell counts, *n* = 9, Fig. 11d) of CTB + neurons were ppGal + . There was a region between the rostral KF and LPB that contained some ppGal + neurons but none of these were CTB + .

### VRC-projecting neurons in the RTN are activated in response to the central respiratory chemoreflex, and most of these neurons are galaninergic

Typically, the basal level of c-Fos expression in the brain is low in the absence of any cellular stimuli. There was no statistically significant difference in c-Fos expression between the RA and AH groups, within VRC-projecting neurons including ppGal + neurons for any of the forebrain regions assessed (Fig. 6d).

In the pons, 10.5% (88 ± 22, total cell counts, *n* = 3) of VRC projecting neurons in the PAG had a basal c-Fos expression in room air which did not change following AH challenge (6.7%, 68 ± 12, total cell counts, *n* = 4) (Fig. 7c, e, f). While CTB + neurons were located only in the DMPAG, LPAG and VLPAG, c-Fos + neurons were distributed across all PAG compartments.

In the KF, activation of CTB + neurons and CTB + /Phox2b + subpopulation did not change from basal levels after AH challenge (3.7% vs 4.1%, 35 ± 4 vs 51 ± 20 total cell counts, *n* = 3–4) and (20.4% vs 12%, 10 ± 2 vs 15 ± 13 total cell counts, *n* = 3–4) respectively (Fig. 8c, e, f). In the KF region, CTB + neurons were abundant in the KF core, whereas c-Fos + and ppGal + neurons were distributed more dorsally, the latter being positioned between the KF and LPB, and again Phox2b + neurons were distributed more medially (Fig. 8a, b).

A similar trend was observed in the LPB; CTB positive neurons in the LPB had a basal activation of 10.9% (72 ± 9, total cell counts, *n* = 5) and remained non-responsive to AH challenge (9.3%, 83 ± 4.2, total cell counts, *n* = 4). The projecting Phox2b + subpopulation was not responsive to AH challenge either; with a basal activation of 27.9% (24 ± 6, total cell counts, *n* = 5) and 22.4% (10 ± 4, total cell counts, *n* = 4) following AH challenge (Fig. 9c, e, f). CTB + , c-Fos + and Phox2b + neurons in the LPB area had a distinctive distribution with CTB + neurons close to the dorsolateral surface followed by c-Fos + neurons more medially and Phox2b + neurons that appeared further medial to the superior cerebellar peduncle (Fig. 9a, b).

The proportion of chemoresponsive NTS neurons projecting to the VRC did not increase significantly following AH challenge (8.2% vs 12.4%, 219 ± 30 vs 429 ± 32 total cell counts, *n* = 4–5). Similar results were obtained for both of the Phox2b + and ppGal + projecting NTS subpopulations (10.1% vs 17%, 172 ± 36 vs 315 ± 44, total cell counts *n* = 4–5) and (6.7% vs 16.9%, 26 ± 2 vs 85 ± 29, total cell counts, *n* = 4–5) respectively (Fig. 11a–c, e–f).

The RTN was the only region that contained VRC projecting neurons exhibiting increased c-Fos immunoreactivity in response to AH challenge. Out of all the RTN neurons projecting to the VRC, 7.8% exhibited basal activation in animals under room air conditions (17 ± 6 total cell counts, *n* = 5), compared to 43.7% (90 ± 5 total cell counts, *n* = 4) (*p* < 0.0001) in animals exposed to AH challenge. In the ppGal + RTN subgroup projecting to the VRC, 3.1% (2 ± 1 total cell counts, *n* = 5) exhibited baseline activation which increased to 54.7% (33 ± 6, total cell counts, *n* = 4) (*p* < 0.0001) in response to AH challenge (Fig. 10a–c, e–f).

## Discussion

**Fig. 12:**
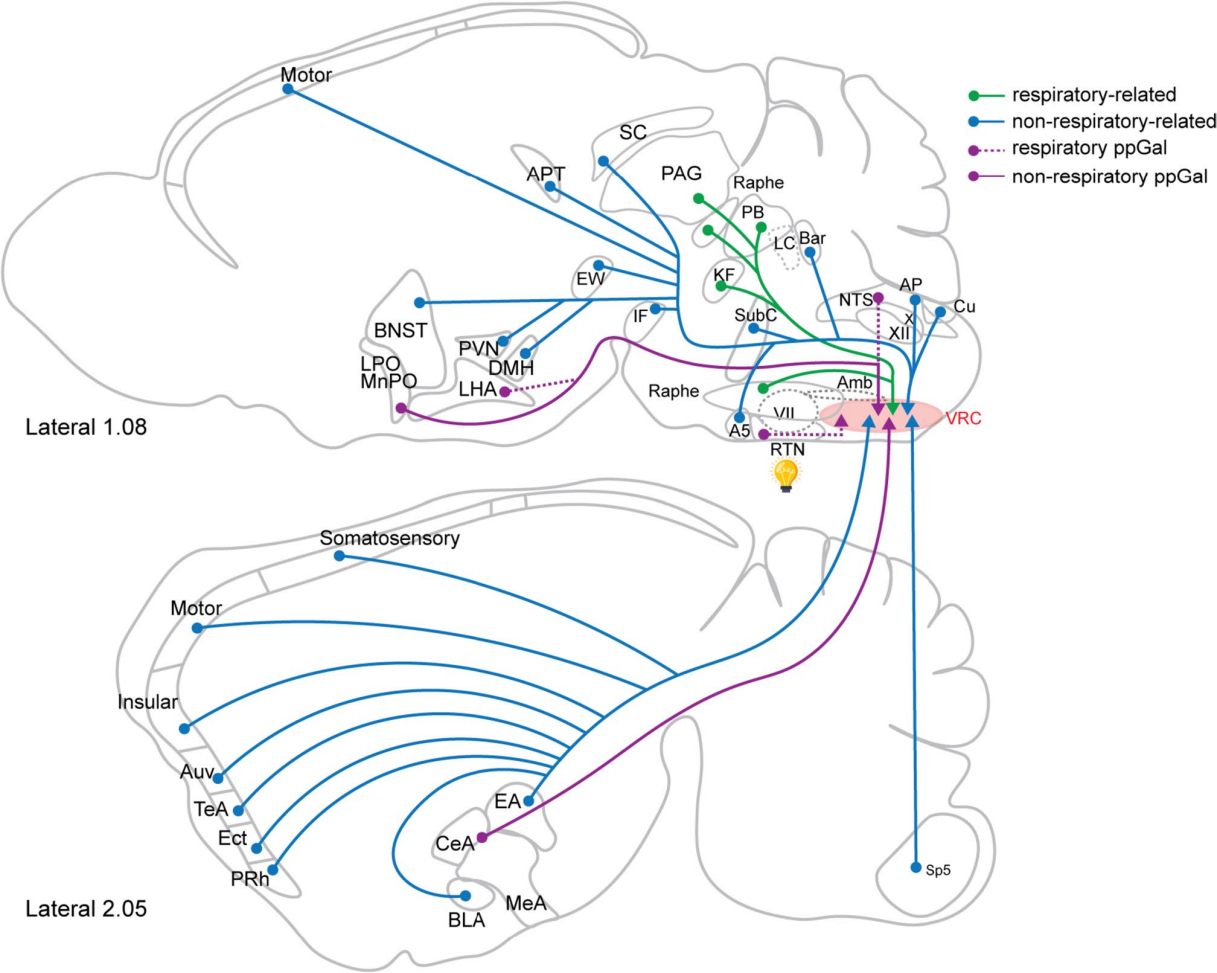
Sagittal schematic diagrams of mouse brain sections at two different lateral levels showing brain sites that provide inputs to the VRC. Green arrows indicate respiratory related, blue arrows indicate non-respiratory-related, purple arrows indicate galaninergic, dashed purple arrows indicate respiratory-related galaninergic projections to the VRC. Only RTN projections, including galaninergic subset, were hypercapnia activated (light bulb). See Table 2 for abbreviations

This study describes all neuronal populations that project to the VRC in the mouse brain (Fig. 12), demonstrating that the VRC receives input from an extensive array of brain regions. Highlighting the importance of peptidergic regulation of breathing, a subset of these VRC-projecting neurons express ppGal mRNA and are chemosensitive (CO_2_-stimulated). Building upon our prior finding of GalR1 mRNA expression in the BötC and preBötC (Dereli et al. 2019), this study further substantiates the potential for a role of galanin within the VRC circuitry in contributing to acute or adaptive responses to hypercapnic stimuli.

### Methodological considerations

There are some technical limitations that need to be taken into consideration. First, the VRC is positioned within a functionally heterogeneous area of the ventrolateral medulla that contains neurons related to cardiovascular, respiratory, chemosensory, metabolic and motor functions. The degree to which neurons subserving different functions are spatially segregated within this region is low, and despite using a relatively small volume of CTB (20 nl), CTB deposition sites encompass multiple autonomic nuclei, including multiple VRC compartments. Furthermore, CTB can be taken up by fibres of passage that traverse the injection site (Saleeba et al. 2019): as a result, CTB-labelled neurons likely denote a projection to the injection site, with the caveat that their post-synaptic targets may include both respiratory and non-respiratory populations. In the future, employing retrograde viral vectors such as AAV or rabies designed to target specific neuronal populations would overcome this problem.

### Neuronal inputs to the VRC

In total there were 30 brain nuclei including 51 subnuclei projecting to the VRC, some of which were identified in previous studies; these include the raphe nuclei (Morinaga et al. 2019; Ribas-Salgueiro et al. 2005), RTN (Rosin et al. 2006; Weston et al. 2004), PVN (Kc et al. 2002; Mack et al. 2007), NTS (Alheid et al. 2011; Ellenberger and Feldman1990; Geerling and Loewy 2006; Nunez-Abades et al. 1993; Otake et al. 1992; Rinaman 2010), SC (Kaneshige et al. 2018) and pontine respiratory group (Ezure and Tanaka 2006; Gang et al. 1995). Our study additionally identified the following populations to have inputs to the VRC: motor, insular, sensory cortex areas, lateral and medial preoptic areas, amygdaloid regions (extended, central, medial, basomedial and basolateral), hypothalamic areas (dorsomedial and lateral), BNST, reticular formation, interfascicular nucleus, anterior pretectal nucleus, PAG (DMPAG, VLPAG, LPAG), Pre-Edinger-Westphal nucleus, Barrington’s nucleus, A5 region, SubC, cuneate nucleus and trigeminal nucleus.

The current study sought to identify the VRC projecting neuronal populations that are activated by acute hypercapnia, indicating their role in CO_2_- stimulated breathing. Our findings revealed that amongst the various CTB + brain regions, only RTN neurons displayed a significant increase in c-Fos immunolabeling following exposure to hypercapnia. This finding sheds light on how the RTN-VRC network operates and also highlights the critical role of the RTN in sensing changes in breathing due to chemical stimuli. Although not responsive to hypercapnia, other VRC-projecting neuronal populations identified in this study are known to have functional contributions to the control of breathing such as the primary motor cortex, SubC, LPB, KF and BNST. Previous studies show that inputs from the motor cortex underlie the voluntary control of respiration (Ozaki and Kurata 2015) and orchestrate tongue movement to allow speech and swallowing (Pouget et al. 2018; Sawczuk and Mosier 2001). Similarly, in newborn mice and sheep, the SubC demonstrates increased activation (c-Fos immunoreactivity) following hypoxia (Joubert et al. 2016; Nitsos and Walker 1999). Pharmacological inhibition and electrical stimulation experiments suggest that A5 neurons modulate the respiratory response evoked from the PB and KF neurons (Dawid Milner et al. 2003). Furthermore, the discharge pattern of BNST neurons in cats suggests that these neurons contribute to the inspiratory onset of the respiratory cycle (Terreberry et al. 1995). Neuronal circuitry underlying these functions was previously not clear. By identifying VRC projecting neurons in these neuronal populations, this study supports previous functional findings and contributes to a deeper understanding of the established literature.

### Galaninergic projections to the VRC

This study identified multiple brain sites that provide convergent galaninergic inputs to the VRC, suggesting that the galanin-related respiratory adaptation could be mediated by diverse inputs that may, depending on the context, be working independently or in parallel.

In the hypothalamus, LPA and MPA neurons provided small numbers of galaninergic inputs to the VRC. Optogenetic studies show that galaninergic MPA neurons govern parenting behaviour in mice (Kohl et al. 2018; Wu et al. 2014); their involvement in the regulation of breathing has not yet been established. Here, preoptic area subdivisions did not exhibit notable c-Fos immunoreactivity following chemoreflex challenge; this suggests that this area is unlikely to be involved in chemoreflex-mediated respiratory effects, although a role for the preoptic area in driving respiratory activity in response to hyperthermia has previously been described (Boden et al. 2000). Furthermore, preoptic inputs have recently been implicated in plastic changes to sympathetic output evoked by chronic intermittent hypoxia (Marciante et al. 2020), and therefore the identification of galaninergic projections from the preoptic area to the VRC supports the involvement of this region in the control of breathing.

In this study, the LHA was another hypothalamic region identified to have inputs to the VRC from a sparse number of galaninergic neurons. The LHA has orexinergic projections to the preBötC, but this represents only 2.9% of all the orexin-containing neurons in the area (Young et al. 2005). Galaninergic projections from the LHA to the VRC represent a similar proportion of neurons (3.3%). Focal acidification experiments have reported some innate chemosensitivity of the LHA (Li et al. 2013), however we observed no induction of c-Fos in CTB-labelled LHA neurons and conclude that LHA neurons that project to the VRC are unlikely to contribute to CO_2_-chemosensitivity.

In the amygdala, CTB-immunoreactive cell bodies were detected in the extended amygdaloid nuclei, CeA, MeA, basomedial and basolateral amygdaloid nuclei. None of these areas were associated with c-Fos induction after hypercapnia, and only the CeA and MeA contain galaninergic neurons, consistent with previous studies (Kuteeva et al. 2004), of which only a small number provide input to the VRC. Deep electrode stimulation of the MeA induces apnoea in temporal lobe epilepsy patients (Nobis et al. 2018) and contributes to blunted CO_2_ responsiveness during neonatal maternal separation (Tenorio-Lopes et al. 2017). Similarly, electrical stimulation of the CeA evokes phrenic nerve discharge in rabbits (Cox et al. 1987). These studies provide evidence that CeA and MeA are involved in the regulation of breathing and, due to the proximity of these areas, electrical stimulation probably activates both subdivisions. Nevertheless, based on the findings of the current study, it seems unlikely that galaninergic CeA/MeA-VRC circuitry contributes to these effects.

Many studies have established that respiratory chemoreceptor populations in the NTS project to the VRC (Alheid et al. 2011; Ellenberger and Feldman 1990; Geerling and Loewy 2006; Nunez-Abades et al. 1993; Otake et al. 1992; Rinaman 2010). This study shows that a subgroup (~ 15%) of NTS projections to the VRC contains ppGal mRNA. However, neither VRC projecting NTS neurons overall nor the galaninergic NTS neurons demonstrated c-Fos immunoreactivity in response to hyperoxic hypercapnia_,_ when compared to baseline c-Fos immunoreactivity. In addition to peripheral chemoreceptors that are highly sensitive to hypoxia, rather than hypercapnia, glutamatergic NTS neurons have previously been demonstrated to be hypoxia-sensitive (Takakura et al. 2006; Kline et al. 2010). Whether the galaninergic subpopulation of VRC projecting NTS neurons are hypoxia-activated remains to be determined.

The RTN has well-established projections to all subdivisions of the VRC (Rosin et al. 2006). This study demonstrates that ~ 44% of VRC-projecting RTN neurons are CO_2_-responsive. Furthermore, an anterograde tracer study has shown galanin immunoreactive boutons on VRC neurons arising from RTN neurons (Bochorishvili et al. 2012). Overall, ~ 32% of VRC-projecting RTN neurons (~ 70 neurons unilaterally) contained ppGal mRNA and ~ 55% of these galaninergic projections were chemoresponsive. Out of all brain regions that project to the VRC, only the galaninergic RTN subset demonstrated significant neuronal activation in response to AH challenge, when compared to room air. This highlights the crucial role of the RTN chemoreceptors, and in particular the galaninergic subpopulation, in CO_2_-stimulated drive to breathing, via direct innervation of VRC neurons.

In conclusion, this study reveals the brain-wide distribution of inputs to the VRC of the mouse. 12 out of 51 VRC-projecting subnuclei were galaninergic; these were the BNST, CeA, MeA, AHN, LPA, MPA, DMH, VMH, PVN, LHA, RTN and NTS. All 12 regions also contained non-galaninergic projections, the neurochemistry of which remains unidentified. We surveyed the extent to which VRC-projecting neurons are activated by acute hypercapnic stimuli and found that only VRC-projecting neurons in the RTN are both galaninergic and activated in response to hypercapnia. Together these results highlight the importance of this RTN-VRC galaninergic circuitry in central respiratory chemoreflex function by showcasing control of breathing under hypercapnic conditions via galaninergic neurons. Further work investigating other VRC projecting areas is required to examine the circumstances under which they are functionally active, their specific neurochemistry and postsynaptic targets of all these projections.

### Supplementary Information

Below is the link to the electronic supplementary material.

Supplementary file1 (DOCX 10349 KB)

## Data Availability

The datasets generated during and/or analysed during the current study are available from the corresponding author on reasonable request.
