# Supplementary material for: Galaninergic and hypercapnia-activated neuronal projections to the ventral respiratory column
Source: Brain Struct Funct. 2024 Apr 5;229(5):1121–42. doi: 10.1007/s00429-024-02782-8 (PMC11147908; doi:10.1007/s00429-024-02782-8)
Supplement: Supplementary file 1 — Supplementary file1 (DOCX 10349 KB) [file 429_2024_2782_MOESM1_ESM.docx]

**Galaninergic and hypercapnia-activated neuronal projections to the ventral respiratory column**

**Supplementary Information**

**Authors**

1. Ayse S. Dereli, Department of Pharmacology, School of Biomedical Sciences, University of New South Wales, Sydney, Australia, [ayse.dereli@uclouvain.be](mailto:ayse.dereli@uclouvain.be) (Corresponding author)
2. Alice Y. S. Oh, Department of Pharmacology, School of Biomedical Sciences, University of New South Wales, Sydney, Australia
3. Simon McMullan, Macquarie Medical School, Macquarie University, Medical School, Sydney, Australia
4. Natasha N. Kumar, Department of Pharmacology, School of Biomedical Sciences, University of New South Wales, Sydney, Australia, [natasha.kumar@unsw.edu.au](mailto:natasha.kumar@unsw.edu.au) (Corresponding author)
5. **Anatomical determination of the neuronal populations investigated**

The RTN, LC and NTS neurons were identified by their Phox2b labelling and neuroanatomical location. In the pons, the LPB was determined to be the region dorsolateral to the subcoeruleus nucleus. The location of the KF was confirmed with reference to the mouse stereotaxic brain atlas (Franklin and Paxinos, 2007). The KF neurons were clearly identified from the surrounding populations by their VRC projecting (CTB labelled) neuronal clusters, as described in the results. Only CTB positive neurons were counted. The PAG was identified by superimposition of the tissue section with the corresponding section in the stereotaxic atlas (Franklin and Paxinos 2007) and cross-referencing with landmarks including inferior colliculus, superior cerebellar peduncle and cerebral aqueduct.

For the hypothalamus, anatomical boundaries were defined as follows: the dorsal boundary was defined as the most central point of the internal capsule; the most lateral point was defined as the medial notch of the ventral surface of the cerebrum; the medial boundary was defined as the vertical line dividing the third ventricle into two equal parts; the ventral boundary was set as the extension of the line drawn from the ventral border of the hypothalamus (**Supplementary**  **Fig. 1**). Amygdala subnuclei delineations were determined according to the distance from bregma (Franklin and Paxinos 2007). Then, the piriform cortex was differentiated from more dorsal structures e.g., perirhinal, insular cortex (**Supplementary**  **Fig. 2**). Cortical-like structures medial to the piriform cortex were then classified according to the atlas (Franklin and Paxinos 2007).


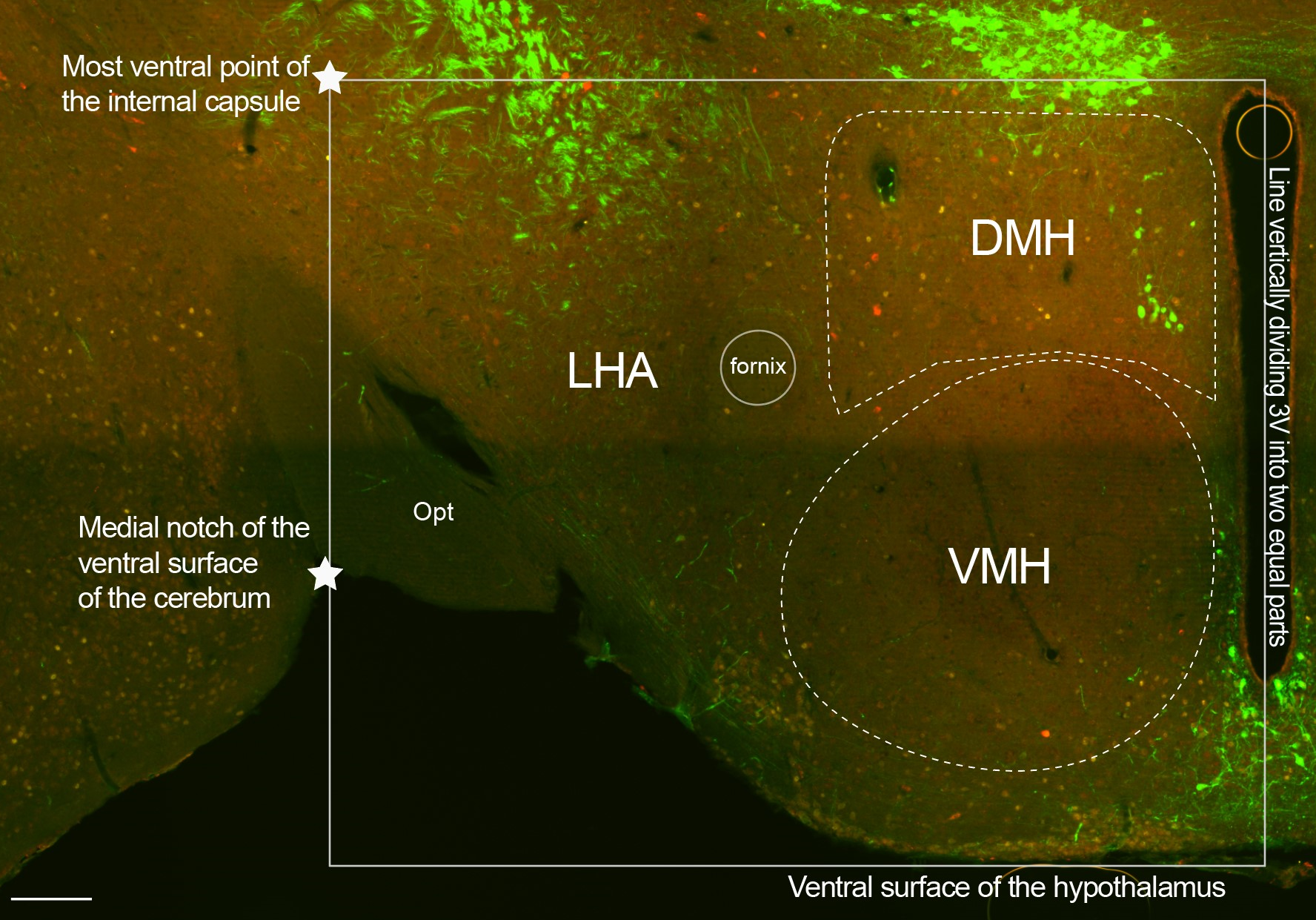


**Supplementary Fig.**  **1** Hemisection view demonstrating the defined boundaries of the hypothalamus. Green: TH ; Yellow: c-Fos ; Red: CTB . Green TH+ cells mark the catecholaminergic cells of ArcH and the A12 dopaminergic cells. Medial border: vertical line dividing the 3V into two equal parts; ventral border: a line drawn extending the ventral surface of the hypothalamus; lateral border: line passing through the medial notch of the ventral surface of the cerebrum, perpendicular to the medial border; dorsal border: a line drawn parallel to the ventral border, passing through the most ventral point of the internal capsule. Opt: Optic tract; 3V: third ventricle; VMH: Ventromedial hypothalamus; DMH: dorsomedial hypothalamus. Scale bar = 500 μm


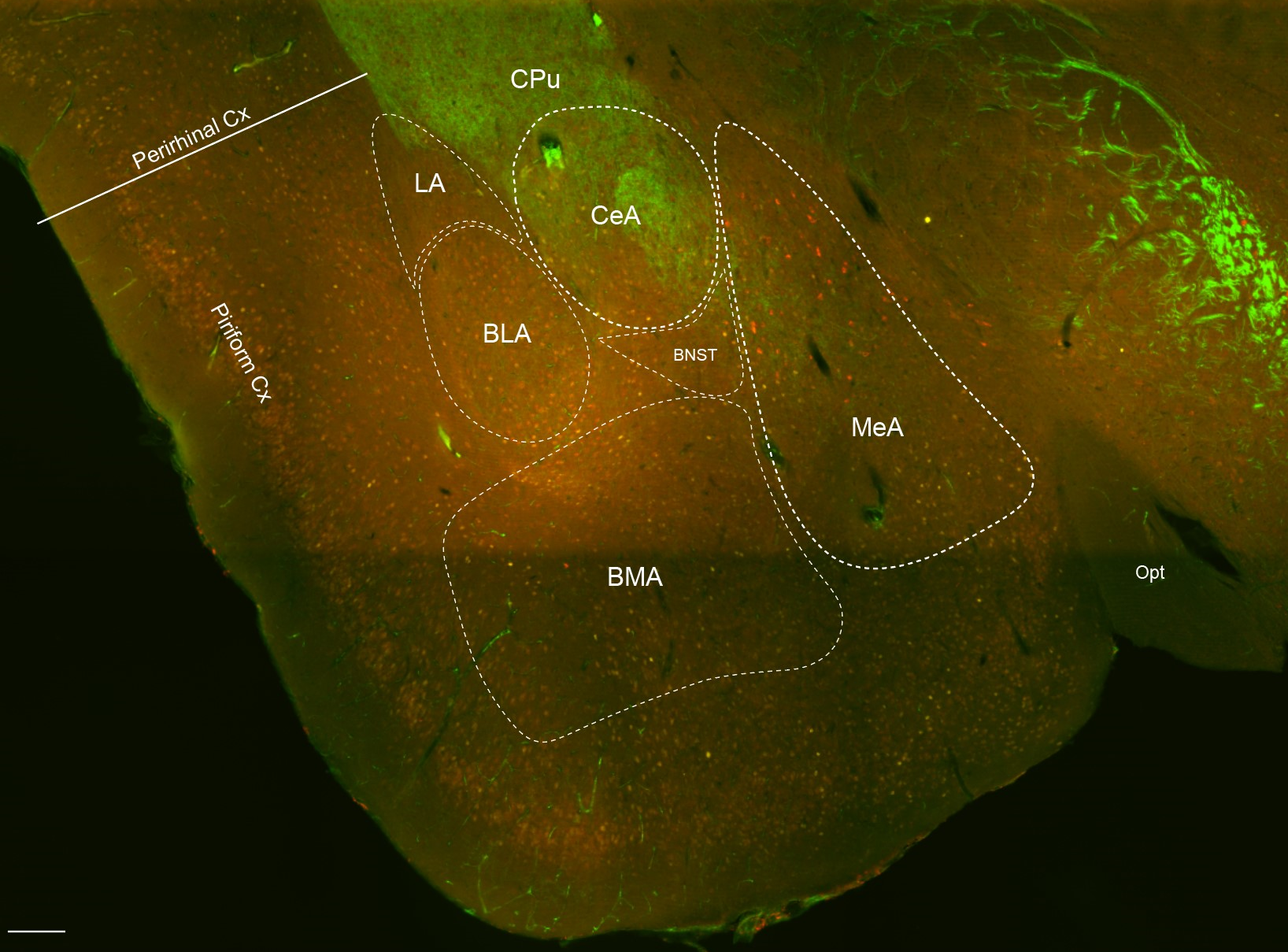


**Supplementary**  **Fig. 2** Exemplary amygdala subnuclei delineations at Bregma = 1.58 mm. Green: TH ; Yellow: c-Fos ; Red: CTB . BLA: basolateral amygdaloid nucleus; BMA: Basomedial amygdaloid nucleus; BNST: Bed nucleus of the stria terminalis; CeA: central amygdaloid nucleus; CPu: caudate putamen; LA: lateral amygdaloid nucleus; opt: Optic tract. Scale bar = 500 μm

1. **Grading for qualitative analysis (Table 2)**


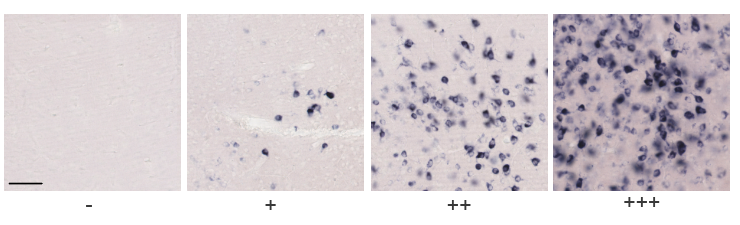


**Supplementary**  **Fig. 3** Representative images illustrating the qualitative analysis scale used to grade ppGal expression. Strong ppGal expression was seen in the cytoplasm of neurons. -: no ppGal expression; +: low expression; ++: moderate expression; +++ high expression. Scale bar = 100 μm

1. **Density of CTB+ neurons ipsilateral vs contralateral side of injection site**


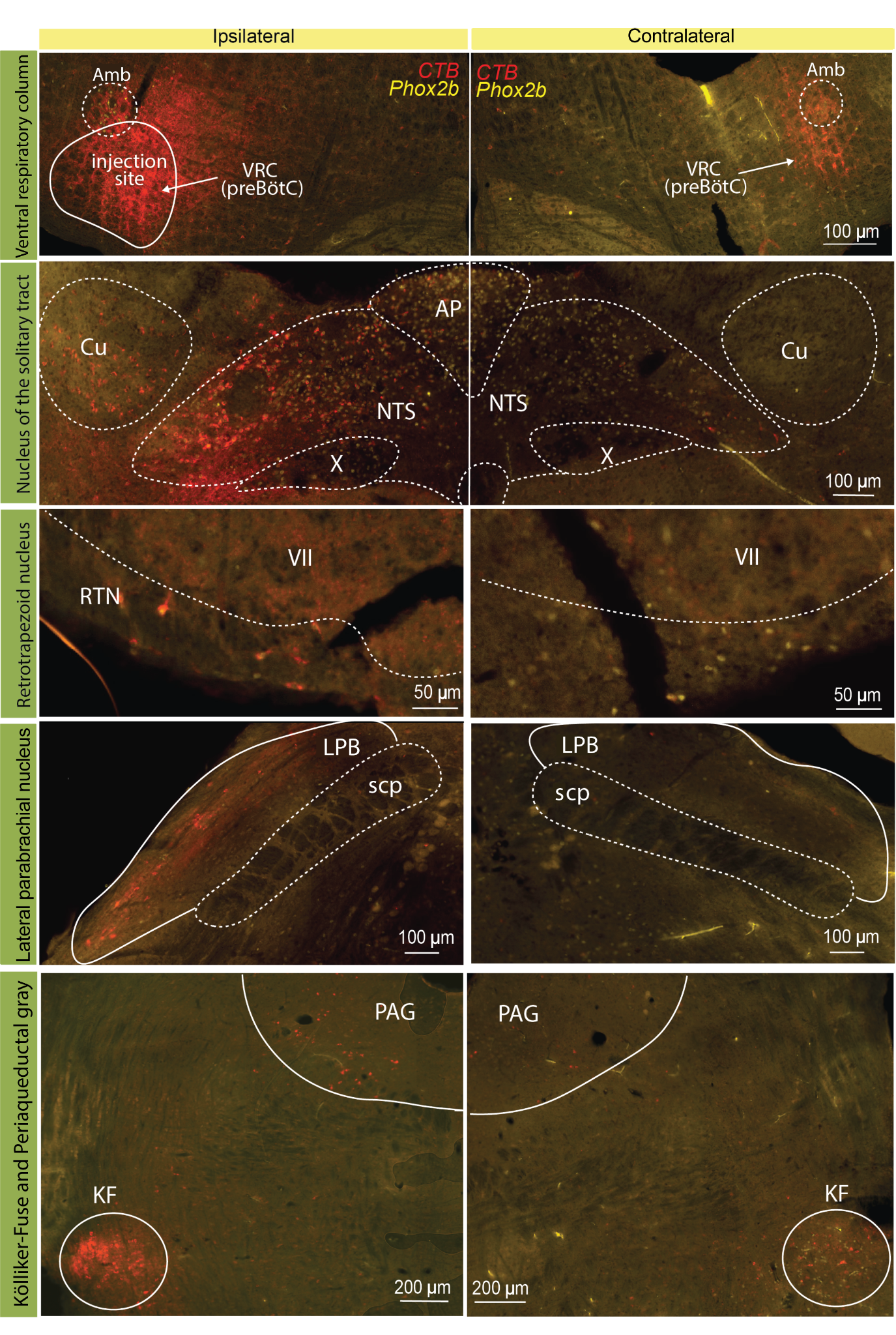


**Supplementary Figure 4** VRC projections predominantly arise ipsilateral to the CTB injection site, including from the nucleus of the solitary tract (NTS), cuneate nucleus (Cu), retrotrapezoid nucleus (RTN), lateral parabrachial nucleus (LPB), periaqueductal gray (PAG) and Kölliker-Fuse (KF). Few VRC projections originate from the side contralateral to the injection site. CTB is in red and Phox2b is in yellow. Other abbreviations: nucleus ambiguus (Amb), preBötzinger complex (preBötC), area postrema (AP), dorsal motor nucleus of the vagus (X), facial motor nucleus (VII), superior cerebral peduncle (scp).
